# Genotype by environment interaction and local adaptation of commercial legume cover crop genotypes

**DOI:** 10.3389/fpls.2026.1903778

**Published:** 2026-08-10

**Authors:** Raksha Thapa, Joel Douglas, John Englert, Heather Dial, Kelly Robbins, Virginia Mae Moore

**Affiliations:** 1 Section of Plant Breeding and Genetics, School of Integrative Plant Science, Cornell University, Ithaca, NY, United States; 2 Retired, Fort Worth, TX, United States; 3 US Department of Agriculture Natural Resources Conservation Service, Plant Materials Program, Washington, DC, United States; 4 US Department of Agriculture (USDA) Natural Resources Conservation Service, Plant Materials Program, Portland, OR, United States

**Keywords:** breeding, cover crops, genotype-environment interaction, legume crops, weather-trait relation, winter hardy, winter survival

## Abstract

Inconsistent winter survival is one of the major challenges in fall-planted legume cover crops. Although several genotypes marketed as winter-hardy are available commercially, their performance is unknown across many US regions. Climate change has further escalated this problem with unpredictable weather conditions. To address this gap, we evaluated winter survival of 30 genotypes of hairy vetch, winter pea, crimson clover, red clover, and balansa clover in 31 US location-years over two growing seasons (2016-2018). We analyzed the effect of genotype, environment, genotype-by-environment interaction, and weather-trait associations. Hairy vetch cultivars ‘CCS Groff’, ‘Purple Prosperity’, and ‘TNT’ showed the highest overall winter survival combined with stable performance across environments. Variance partitioning indicated that environment (43.2%) and genotype-by-environment interaction (35.9%) explained more variation in winter survival than the genotype main effect (17.1%), indicating that cultivar performance was considerably context-dependent and that environment-specific recommendations are warranted. In most environments, winter survival was moderate (0.50-0.80) to high (greater than 0.80), except in two site-years where even the superior genotypes had less than 0.5 winter survival, and substantial year-to-year variation occurred even within the same locations. Environment-level correlation analysis indicated that temperature in November, February, and March (positive effect) and snowfall in these same months (negative effect) were most consistently associated with winter survival, though no single weather variable or combination dominated, reflecting the complexity of winter survival as a trait. The substantial genotype-by-environment interaction observed, together with very low winter survival in some environments, highlights the necessity of multi-environment testing and developing more winter-hardy and stable legume cover crop cultivars.

## 1 Introduction

Growing interest in soil health and the push for sustainable agricultural practices have renewed attention towards cover crops, plants cultivated primarily for their ecological benefits. Cover crops can play a critical role in improving soil structure, enhancing nutrient cycling, preventing erosion, and suppressing weeds. In 2022, cover crops were planted on 18 million acres, representing about one-fifth the acreage planted to corn, one-fifth of that planted to soybean and slightly more than one-third of that planted to wheat (USDA NASS, 2024). Among cover crop species, legumes such as winter pea (*Pisum sativum* L.), hairy vetch (*Vicia villosa*), and three clover species (crimson clover*,Trifolium incarnatum*; red clover, *Trifolium pratense*; and balansa clover, *Trifolium michelianum*), are commonly planted for their nitrogen-fixing ability, in addition to their contributions to soil health improvement and weed suppression (SARE-CTIC, 2016, 2017, 2020, 2023). Genotypes representing all five species were evaluated in this study. Inconsistent winter survival is a major challenge for usage of fall-planted (e.g., winter annual or short-lived perennial) legume cover crops such as winter pea, hairy vetch, and clover species. While these species typically perform well in warmer southern regions, their survival in northern environments is highly variable across locations and years. Poor winter survival reduces spring biomass production, limits ground cover, and ultimately diminishes the ecological benefits the cover crop is intended to provide, including biological nitrogen fixation, weed suppression, erosion control, and carbon sequestration (Florence et al., 2019; Kaye et al., 2019; Ranaldo et al., 2020; Thurston et al., 2022).

Winter survival is influenced by environmental conditions, genotypes, and genotype-by-environment (G×E) interactions. Even within the same geographic location, weather conditions can vary dramatically from year to year. In addition to weather, soil properties and other environmental factors, such as soil texture, drainage, soil moisture, organic matter, and topography may differ within the same planting site. Among weather parameters, winter temperature and snow cover are considered the most influential. Prolonged exposure to freezing temperatures generally reduces survival (Zheng et al., 2018), whereas snow often provides thermal insulation that buffers crowns and root systems from extreme soil temperature fluctuations (Drescher and Thomas, 2013). Furthermore, climate change is altering historical weather patterns. Traditionally warmer regions are experiencing unusual cold extremes, while northern areas face shifting winter conditions, including frequent freeze–thaw cycles (Qian et al., 2024; Monge-Sanz, 2024). More frequent freeze–thaw cycles can cause direct physical damage to plant tissue and frost heaving of the soil, increasing the risk of winterkill in legume cover crops. Climate warming is also projected to reduce snowfall and snow cover duration and to alter the timing of fall hardening and winter thaw cycles, which may increase the risk of winter damage to overwintering forage and cover crops (Qian et al., 2024). Because snow cover and freeze–thaw frequency are also the parameters most protective of (or damaging to) winter survival, these climate-driven shifts are especially consequential: regions losing their historical snow cover, or gaining unaccustomed freeze–thaw exposure, may see genotypes that were once reliably productive at a site may no longer perform consistently. This increases environmental variability across years within the same location, compounding the challenge of predicting genotype performance.

Genotype is another key factor affecting winter survival. Currently, numerous winter legume cover crop genotypes are available on the market, many of which are marketed as winter hardy. However, only a few studies have tested a subset of these cultivars, and only at a limited number of locations. For example, Casey and Spellman (2020) and Herget (2020) evaluated some cultivars of these species in Pullman, WA and Elsberry, MO, respectively. Vann et al. (2021) evaluated 18 pea cultivars, one crimson clover cultivar, and one hairy vetch cultivar in Beltsville, MD, Clayton, NC, and Kinston, NC. Thus, a comprehensive study evaluating these legume cultivars across multiple environments in the US is lacking, and their actual hardiness across diverse environments remains unknown. This gap is particularly important given that substantial variation exists among legume cover crop genotypes under contrasting environments (Moore et al., 2020; Boukrouh et al., 2024, 2023). Moreover, these genotypes can interact with the environment, performing differently across sites and years. G×E has been shown to influence performance in several species and traits (Thomas et al., 1993; Okii et al., 2018; Secchi et al., 2021). Multi-environment trials are essential for quantifying G×E, providing local recommendations, and identifying genotypes that are both high-performing and stable (i.e., able to maintain relatively consistent performance across diverse environment.

To address these challenges, a two-year multi-environment trial was conducted from 2016 to 2018 across 19 of the United States Department of Agriculture Natural Resources Conservation Service (USDA-NRCS) Plant Materials Center locations. The experiment evaluated 30 genotypes representing five common winter legume cover crop species with abundant seed availability and popularity among farmers. These locations spanned a broad geographic range across the United States, from east to west and north to south, representing the most geographically extensive evaluation of winter legume cover crop genotypes conducted to date. The objectives of the study were to:

- Identify superior genotypes for each environment and provide local recommendations
- Identify the most stable genotypes and overall best performers across environments
- Identify weather factors influencing winter survival

## 2 Materials and methods

### 2.1 Materials and environment

**Figure 1:**
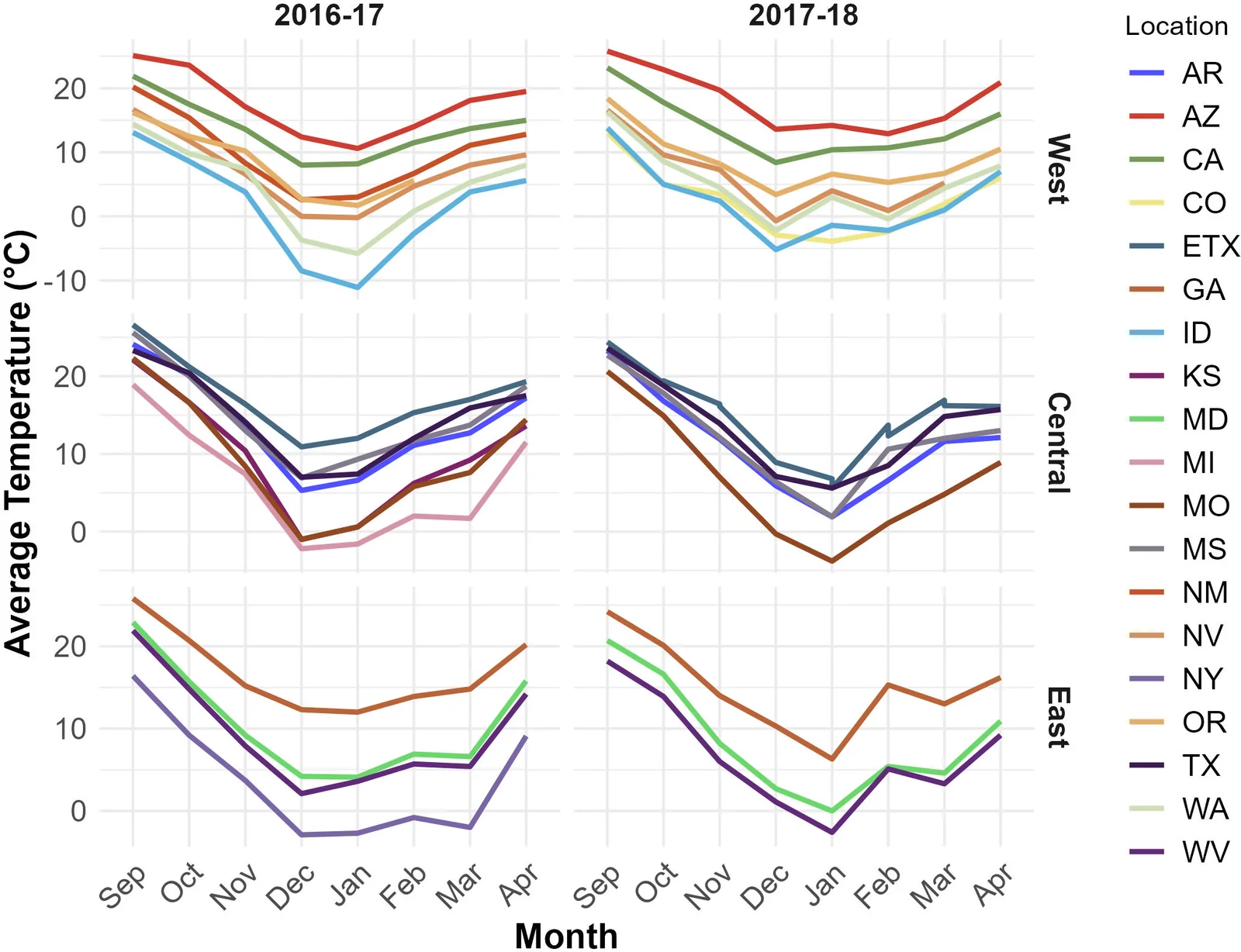
Monthly average temperature (°C) across the environments of the multi-environment trial, shown by region (West, Central, East; rows) and growing season (2016–2017, 2017–2018; columns). Each line represents one environment, coded by state; the x-axis spans September–April.

**Figure 2:**
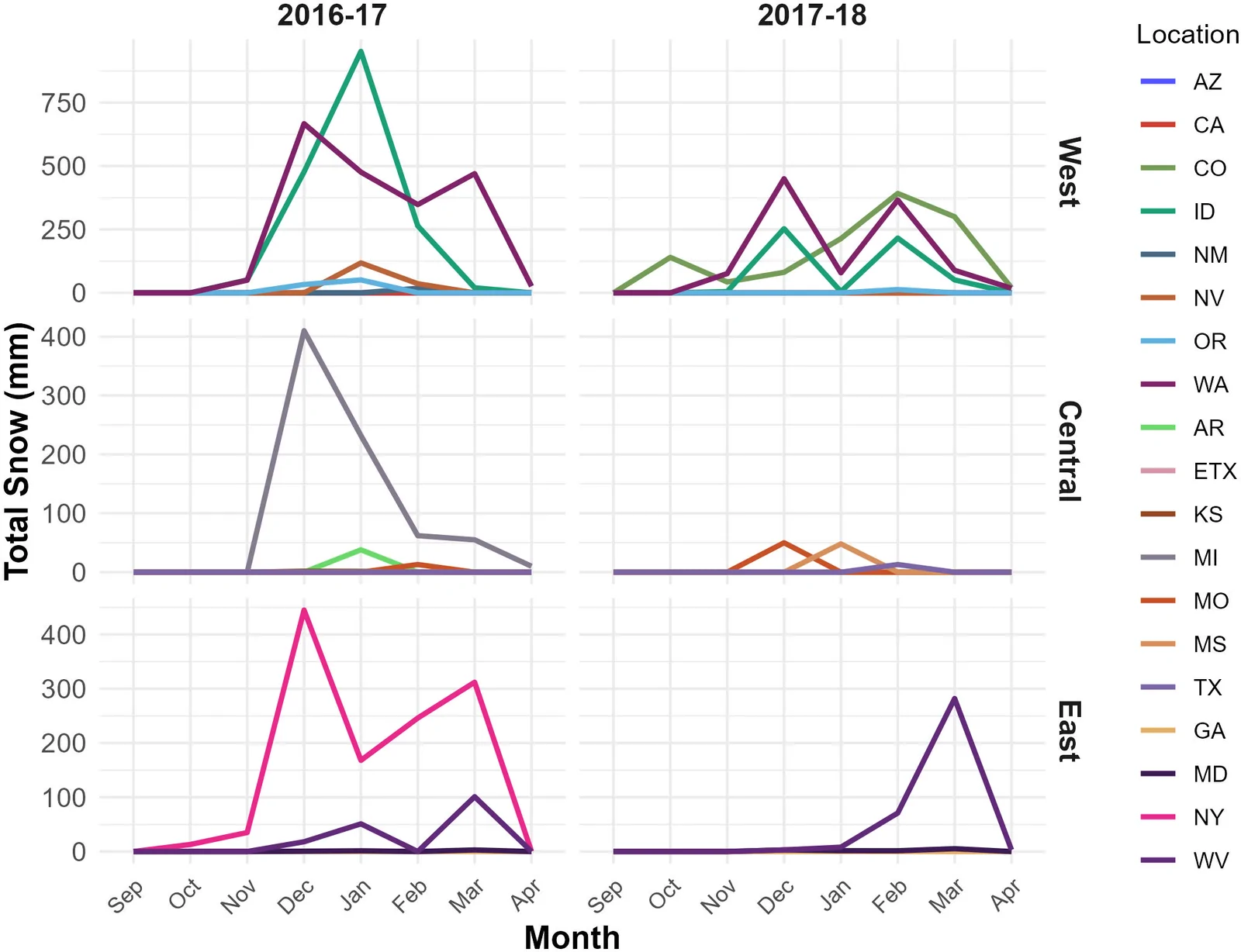
Total monthly snow (mm) across the environments of the multi-environment trial, shown by region (West, Central, East; rows) and growing season (2016–2017, 2017–2018; columns). Each line represents one environment, coded by state; the x-axis spans September–April.

This study was conducted over a two-year period, from 2016 to 2018. Cover crop treatments were planted in the fall of 2016 and 2017, with data collected from fall through the following spring. Each commercially available cultivar of selected cover crop species (hairy vetch, crimson clover, winter pea, red clover, and balansa clover) (**Table 1**) was purchased from a single source for use at all test locations for both years. Cultivars were planted at the average recommended seeding rates from NRCS cover crop standards in 30–31 location-years (hereafter referred to as environments; details in **Table 2**). Each environment was represented by the final 2 digits of the planting year and the state abbreviation (e.g., 16AR for the Booneville, AR location planted in 2016, see **Table 2**). Three cultivars were planted in only 30 environments because of seed availability; winter pea Survivor 15 was not planted in 17CO, winter pea ‘Dunn’ was not planted in 16WV, red clover ‘Kenland’ was not planted in 16WA (**Tables 1**, **2**). Average monthly temperature and total snowfall are shown in **Figures 1**, **2**, respectively. Soil type for each location is provided in **Table 3**.

**Table 1:** List of species and cultivars, and the number of environments in which each cultivar was tested.

Scientific name	Common name	Cultivar	No. of environments tested
*Pisum sativum*	Spring pea	Arvica 4010	31
		Dunn	30
		Maxum	31
	Winter Pea	Frost Master	31
		Whistler	31
		Windham	31
		Lynx	31
		Survivor 15	30
*Trifolium incarnatum*	Crimson clover	AU Robin	31
		AU Sunrise	31
		AU Sunup	31
		Contea	31
		Dixie	31
		Kentucky Pride	31
*Trifolium michelianum*	Balansa clover	Fixation	31
		Frontier	31
*Trifolium pratense*	Red clover	Cinnamon Plus	31
		Cyclone II	31
		Dynamite	31
		Freedom	31
		Kenland	30
		Mammoth	31
		Starfire II	31
		Wildcat	31
*Vicia villosa*	Hairy vetch	CCS-Groff	31
		Lana^+^	31
		Purple Bounty	31
		Purple Prosperity	31
		TNT	31
		Villana	31

^+^ Lana is a cultivar of woollypod vetch (*Vicia villosa* ssp. dasycarpa), a subspecies of *Vicia villosa*, and was included in this study because of its similarity in usage to hairy vetch.

All plant materials used were commercially available at the time of this study. Most are true cultivars, but some may be named selections from a seed producer. For this manuscript they are all labelled as cultivars.

**Table 2:** Number of genotypes evaluated in each environment in multi-environmental trials.

S.N.	Year	Environments	Location	No of genotypes	Planting date
1	2016	16AR	Booneville, AR	30	10/5/2016
2	2017	17AR	Booneville, AR	30	9/27/2017
3	2016	16AZ	Tucson, AZ	30	10/18/2016
4	2017	17AZ	Tucson, AZ	30	10/18/2017
5	2016	16CA	Lockeford, CA	30	11/8/2016
6	2017	17CA	Lockeford, CA	30	10/19/2017
7	2017	17CO	Meeker, CO	29	8/4/2017
8	2016	16ETX	Nacogdoches, TX	30	10/4/2016
9	2017	17ETX	Nacogdoches, TX	30	10/27/2017
10	2017	17GA	Americus, GA	30	11/3/2017
11	2016	16ID	Aberdeen, ID	30	8/25/2016
12	2017	17ID	Aberdeen, ID	30	8/17/2017
13	2016	16KS	Manhattan, KS	30	9/20-9/22/2016
14	2016	16MD	Beltsville, MD	30	9/16/2016
15	2016	16MI	East Lansing, MI	30	9/16/2016
16	2016	16MO	Elsberry, MO	30	9/12/2016
17	2017	17MO	Elsberry, MO	30	9/15/2017
18	2016	16MS	Coffeeville, MS	30	10/14/2016
19	2017	17MS	Coffeeville, MS	30	10/6-10/11/27
20	2016	16NM	Los Lunas, NM	30	9/27/2016
21	2016	16NV	Fallon, NV	30	10/19/2016
22	2017	17NV	Fallon, NV	30	9/21/2017
23	2016	16NY	Big Flats, NY	30	9/15/2016
24	2016	16OR	Corvallis, OR	30	9/30/2016
25	2017	17OR	Corvallis, OR	30	9/18/2017
26	2016	16TX	Knox City, TX	30	10/4/2016
27	2017	17TX	Knox City, TX	30	10/11/2017
28	2016	16WA	Pullman, WA	29	9/21/2016
29	2017	17WA	Pullman, WA	30	9/26/2017
30	2016	16WV	Alderson, WV	29	9/22/2016
31	2017	17WV	Alderson, WV	30	9/20/2017

**Table 3:** Soil type of the locations evaluated in multi-environmental trails.

Location	Soil type
Aberdeen, ID	Declo silt loam
Alderson, WV	Lobdell loam; Monongahela silt loam
Americus, GA	Orangeburg loam sand
Beltsville, MD	Downer-Hammonton complex; Christiana-Downer complex
Big Flats, NY	Tioga/Unadilla silt loam
Booneville, AR	Leadvale silt loam
Coffeeville, MS	Oaklimeter silt loam
Corvallis, OR	Willamette silt loam
East Lansing, MI	Wasepi sandy loam
Elsberry, MO	Menfro silt loam
Fallon, NV	Sagouspe loamy sand
Knox City, TX	Altus fine sandy loam
Lockeford, CA	Columbia and Vina fine sandy loam
Manhattan, KS	Belvue silt loam
Meeker, CO	Work loam
Nacogdoches, TX	Woden fine sandy loam
Los Lunas, NM	Bluepoint loamy fine sand
Pullman, WA	Palouse silt loam
Tucson, AZ	Comoro silt loam

### 2.2 Field design and preparation

The experiment was conducted over two growing seasons, with different fields used each season at each location. A restricted randomized design with four replications was used across all environments. Within each replication, species were grouped together, and genotypes were randomized within each species (**Supplementary Figure 1**). Species groups were randomized between replications. Details of the planter used, plot size, and fertilizer applications are provided in **Table 4**. Fertilizers were applied according to soil test results to maintain nutrient levels within the medium recommended range. Seeds were inoculated with rhizobia and sown into a well-prepared seedbed. Seeding rates were 65, 667, 614, 603, and 75 plants m^-^² for peas, crimson clover, balansa clover, red clover, and hairy vetch, respectively. Irrigation was applied only in locations where it is considered standard agronomic practice. Weed management was carried out through hand rouging or the application of selective or non-selective herbicides, tailored to site-specific conditions and crop sensitivity.

**Table 4:** Planting equipment, plot dimensions, and fertilizer regimes across trial locations.

Location	Planter used	Plot dimensions (m)	Fertilizer regime (kg/ha)
Aberdeen, ID	Almaco cone plot planter (spacing not reported)	1 row of 6.10 m	Year 1: 80 N, 56 P, 34 K, 56 S Year 2: 45 N, 56 P, 34 K, 56 S.
Alderson, WV	No data	No data	67 P, 34 K.
Americus, GA	Kincaid Plot Seed Drill, 19.1 cm spacing	1.83 × 3.66 m^2^	Medium soil P and K maintained.
Beltsville, MD	Cone seeder, 15.2 cm spacing	0.91 × 3.05 m^2^	45 N, 67 P, 34 K annually.
Big Flats, NY	No data	No data	67 P, 34 K.
Booneville, AR	Kincaid cone seeder, 20.3 cm spacing	1.52 × 3.05 m^2^	67 P, 34 K.
Coffeeville, MS	Kincaid cone seeder, 20.3 cm spacing	1.52 × 7.62 m^2^ (Year 1); 1.52 × 4.57 m^2^ (Year 2)	Year 1: 34 P, 67 K, no N. Year 2: 56 P, 56 K.
Corvallis, OR	Hege cone seeder, 15.2 cm spacing	1.52 × 6.10 m^2^	No fertilizer applied.
East Lansing, MI	No data	No data	67 P, 34 K.
Elsberry, MO	Haldrup no-till drill, 20.3 cm spacing	3.05 × 6.10 m^2^	45 N, 34 P, 67 K.
Fallon, NV	Kincaid cone seeder, 20.3 cm spacing	1.83 × 3.05 m^2^	45 N, 34 P, 67 K only in Year 2.
Knox City, TX	Kincaid cone planter, 101.6 cm spacing	1 row × 7.62 m^2^	67 N, 45 P.
Lockeford, CA	Great Plains Cone Seeder, 17.8 cm spacing	7.62 × 1.52 m^2^	No fertilizer reported.
Manhattan, KS	Kincaid Cone Drill (spacing not reported)	1.52 × 6.10 m^2^	No fertilizer reported.
Nacogdoches, TX	Hege Model 1000 cone planter, 20.3 cm spacing	1.01 × 10.67 m^2^	73 P, 73 K.
Pullman, WA	Kincaid cone seeder, 19.1 cm spacing	1.68 × 3.05 m^2^	No fertilizer reported.
Tucson, AZ	Kincaid cone planter (spacing not reported)	No data	22 N, 22 P (monoammonium phosphate).

### 2.3 Data collection

#### 2.3.1 Plant phenotyping

To evaluate winter survival, plant counts were conducted prior to winter dormancy and following spring regrowth. Fall counts were conducted 28 days after planting or immediately before the first frost. A yardstick or 0.91 m measuring tape was placed alongside the planted row. The presence of a plant at each 2.54 cm increment was recorded. In cases where multiple seedlings occurred within a single increment, they were counted as one plant to standardize density among different species and assess a functional estimate of soil cover and survival. Following winter dormancy, plant survival was assessed within 2–3 weeks of the recorded regrowth initiation date. The same 0.91m section was revisited, and a measuring tape was placed adjacent to the row. The presence of surviving plants at each 1-inch increment was recorded. Additional data were collected on germination, onset of regrowth, plant height, bloom initiation and duration, as well as disease and insect resistance. However, these parameters are beyond the scope of this article (PRISM Climate Group, 2024).

2.3.2 Weather data: Weather data were obtained for each USDA PMC trial location using the zip code of each PMC site. Data were drawn from two sources: the PRISM Group (PRISM Climate Group, Oregon State University; AN91m dataset, 4 km spatial resolution, time series generated September 2024) and the National Oceanic and Atmospheric Administration (NOAA) Global Historical Climatology Network-Daily (GHCN-Daily) dataset, supplemented by the NOAA Applied Climate Information System (ACIS) for monthly and seasonal snowfall totals where GHCN-Daily snowfall records were incomplete. NOAA GHCN-Daily station data nearest to each PMC location served as the primary source; PRISM gridded estimates (extracted at the corresponding location) were used to fill gaps where NOAA station data were unavailable. Daily NOAA records on average, minimum, and maximum temperatures, as well as total monthly snow accumulation were aggregated to monthly values and merged with PRISM data in R, with manual gap-filling using PRISM data performed in Excel.

### 2.4 Data analysis

Data analysis was conducted in R Studio. Winter survival was calculated as:


$$Winter\;survival=\frac{Spring\;count}{Fall\;count}\;$$

#### 2.4.1 Data filtering

Several filtering criteria were applied prior to analysis. The environment 16GA, which included only six genotypes, was excluded due to insufficient representation.

Environments with fewer than three replications per genotype were also removed to maintain statistical rigor. Furthermore, 17AZ location was excluded due to 100% mean survival and 0 variance across genotypes (**Supplementary Table 1**) as this represented extremely favorable condition with no genotype differentiation.

#### 2.4.2 G×E modeling

**Figure 3:**
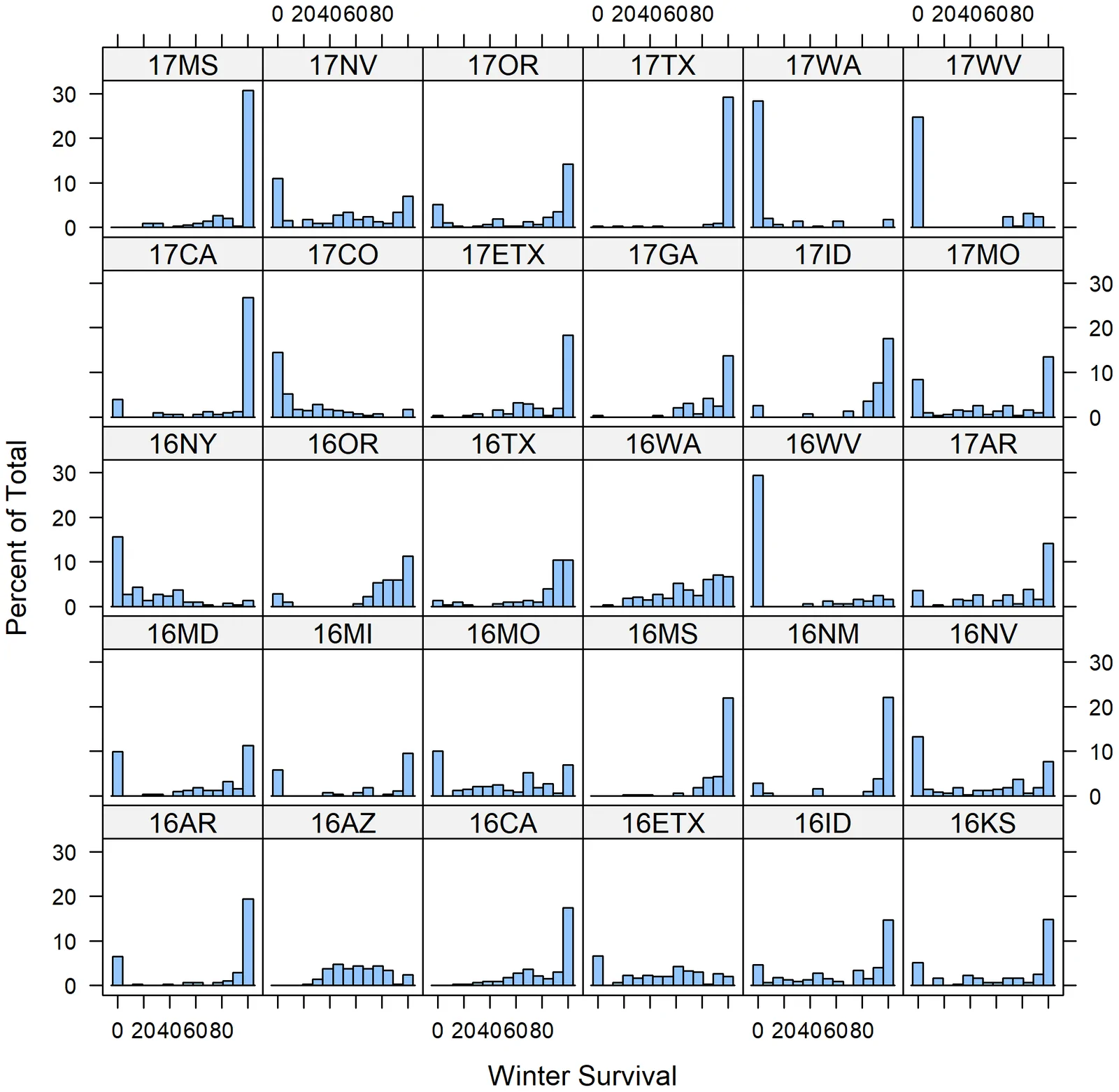
Distribution of the percent winter survival within each environment of the multi-environmental trial.

Winter survival, was highly skewed (**Figure 3**) and attempts to normalize the distribution using logit transformation were unsuccessful.

Given that winter survival values were bounded between 0 and 1, a generalized linear mixed model with a binomial distribution was implemented using the ASReml-R v.4 (Butler et al., 2023). Spring counts were modelled as binomial outcomes with fall counts as the denominator. Prior to model fitting, spring counts exceeding fall counts (likely due to spring emergence of dormant seeds and tillering that confused the count) were corrected by setting spring count equal to fall count for affected plots, resulting in a survival value of 1 (100% survival) for those observations. This affected 506 plots (16.6% of total observations). Fixed effects included environment and genotype, while random effects included interactions between environment and genotype, environment and replication, and environment and replication and species (Equation 1).

(1) $$Loɡit\left(Y_{ijk}\right)=µ+a_{j}+\beta_{i}+u_{ij}+v_{jk}$$

Where, $Loɡit\left(Y_{ijk}\right)$ is the number of plants surviving in spring out of n_ijk_ fall counts for genotype i, environment j, replicate k

*μ* = overall intercept

*a_j_* = fixed effect on the j-th environment

*β_i_* = fixed effect of the i-th genotype $u_{ij}\sim N\left(0,\;\left[\begin{array}{cccc} \sigma_{GE_{1}}^{2} & 0 & \ldots & 0\\ 0 & \sigma_{GE_{2}}^{2} & \ldots & 0\\ \vdots & \vdots & \ddots & \vdots \\ 0 & 0 & \ldots & \sigma_{GE_{n}}^{2} \end{array}\right]\right)\;$ = random effect of G×E for cultivar i in j-th environment with heterogeneous GXE variance per environment. $v_{jk}\sim N\left(0,\sigma_{Rep}^{2}\right)$ = random effect of replication k within environment j

Genotype-by-environment interaction effect was defined in a way that allowed for distinct variance components across environments for genotype effects (chosen over a homogeneous variance model based on lower AIC and BIC values and existence of GXE). This model structure accommodated heterogeneity across environments and appropriately reflected the nested design of the experiment. Best linear unbiased Predictions (BLUPs) were generated for each genotype-by-environment combination and genotypes were ranked for each environment based on BLUPs.

To assess whether the capping decision influenced model outcomes, a sensitivity analysis was conducted by refitting the model after excluding all plots where spring count exceeded fall count, while applying the same minimum replication threshold (n ≥ 3 observations per genotype-environment combination) and same model (Eqn1). BLUPs from the capped and uncapped models were highly correlated across all genotype-environment combinations (Pearson r = 0.963, 95% CI: 0.958–0.968; Spearman ρ = 0.930; n = 900 genotype × environment combinations), indicating that the capping decision did not materially affect genotypic predictions or subsequent stability analyses. The variance heterogeneity factor (deviance/df = 2.09) indicated moderate overdispersion, which is common in binomial GLMMs fitted to field count data; this was partially accounted for by the heterogeneous G×E variance structure, and model stability was confirmed by sensitivity analysis.

BLUPS generated from the GXE model were used to run Finlay-Wilkinson (Finlay & Wilkinson, 1963) stability analysis. BLUPs were used rather than raw means because shrinkage estimation generally improves accuracy by reducing mean-squared error, even when it introduces some bias (Piepho et al., 2008). To prevent genotypes with less supporting data from exerting disproportionate influence on the environmental index, the environmental mean for each location-year was calculated as a fall-count-weighted average of cultivar BLUPs. This approach is conceptually aligned with weighted estimation methods proposed for related genotype-by-environment models, which similarly account for differential reliability of genotype-environment estimates rather than treating all observations as equally informative (Hadasch et al., 2018).

To partition phenotypic variance, a separate variance components model was fitted with all effects, environment, cultivar, G×E interaction, and replicates nested within environment, treated as random (Equation 2). Variance components were estimated on the logit (latent) scale.

(2) $$Loɡit\left(Y_{ijk}\right)=µ+e_{j}+g_{i}+u_{ij}+v_{jk}$$

Where, $Loɡit\left(Y_{ijk}\right)$ is the number of plants surviving in spring out of n_ijk_ fall counts for genotype i, environment j, replicate k

*μ* = overall intercept

$e_{j}\sim N\left(0,\sigma_{Env}^{2}\right)$ = random effect on the j-th environment

${ɡ}_{i}\sim N\left(0,\sigma_{G}^{2}\right)$ = random effect of the i-th genotype

*u_ij_* ~ *N*(0, σ²_GE_) = random effect of G×E for cultivar i in j-th environment

$v_{jk}\sim N\left(0,\sigma_{Rep}^{2}\right)$ = random effect of replication k within environment j

The proportion of variance explained by each source was calculated as the variance component of that source expressed as a percentage of the total estimated biological variance. The total estimated biological variance was obtained by summing the variance components due to environment, genotype, genotype × environment interaction, and replication within environment. Environmental clustering was initially attempted using AMMI, GGE, and hierarchical clustering approaches, but did not yield distinct or biologically meaningful environment groups (see Supplementary Methods for details).

#### 2.4.3 Weather-trait relationships

Temperature and snowfall variables, which are known to affect winter survival performance and have readily available data, were selected as weather parameters. Soil parameters were not included due to the lack of exact field coordinates for each trial location, which precluded extraction of site-specific soil data. For each environment, a single winter survival value was calculated as the count-weighted pooled proportion of surviving plants (total spring count divided by total fall count across all plots within that environment), and its relationships with monthly and seasonal weather parameters were examined using Pearson correlation with Benjamini-Hochberg correction for multiple comparisons across 38 weather variables (n = 30 environments). To account for multicollinearity among weather variables, a multiple regression of environment-level survival on the first four principal components was also fitted; variance inflation factors were then used to verify that the components were not collinear.

## 3 Results

### 3.1 Genotype performance

**Figure 4:**
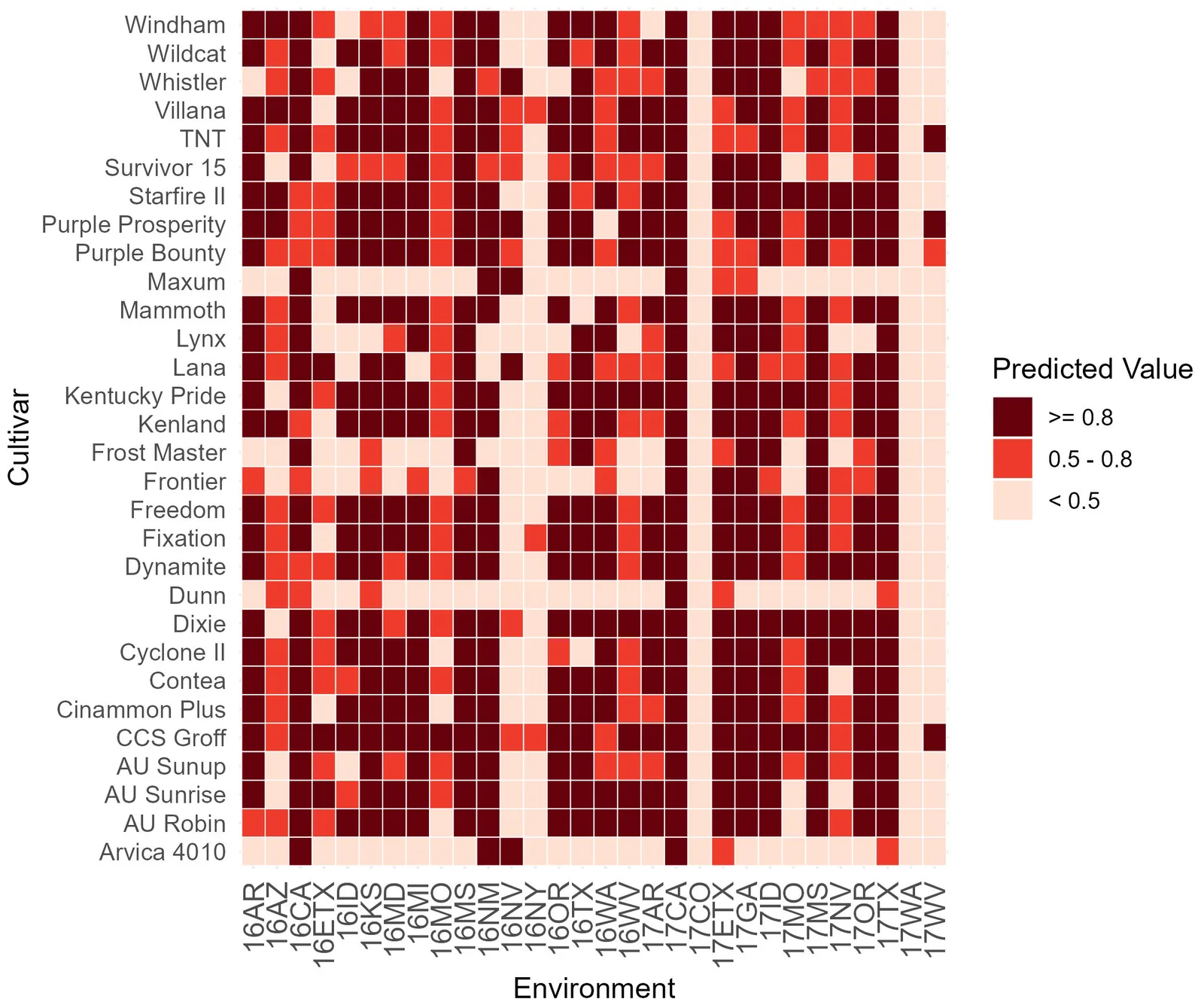
Predicted winter survival of 30 legume cultivars across 30 environments in a multi-environmental trial. For ease of interpretation and visualization, winter survival is divided into three groups (≥ 0.8, 0.8-0.5,<0.5). Higher winter survival is represented by darker color.

**Figure 5:**
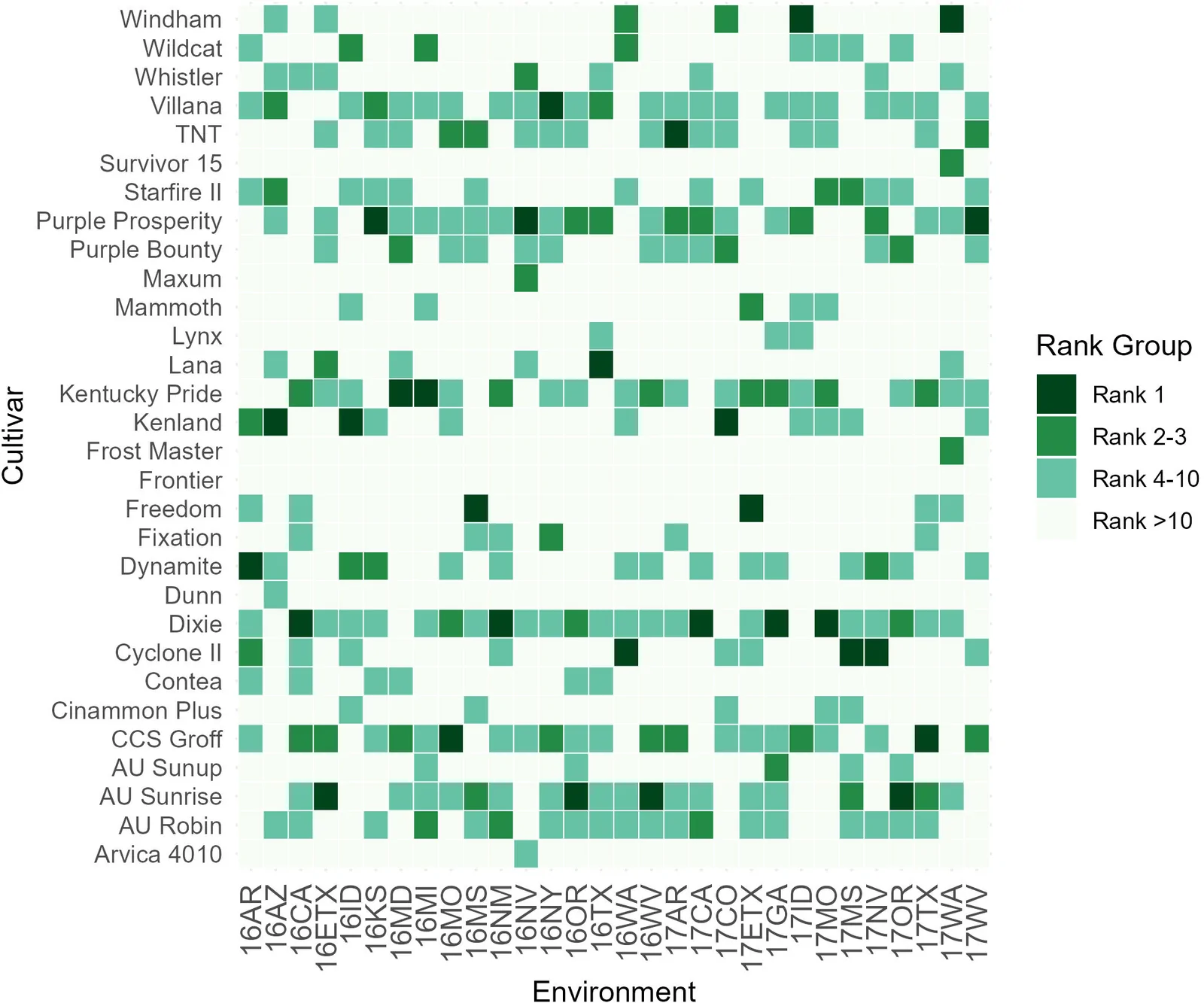
Cultivar ranking across environments based on the predicted winter survival. Higher ranked cultivars are represented by darker color.

Genotypes and environments significantly affected winter survival (P-Value<0.001). BLUPs were generated for each environment × genotype combination (**Supplementary Table 2**; **Figure 4**), and the superior genotypes in each environment were identified (**Figure 5**). Of the 30 environments (total environments after filtering), 27 recorded at least 0.8 winter survival for their best-performing genotype (**Figure 4**). 17WA and 17CO exhibited the poorest survival, with even the best-performing genotypes showing less than 0.5 winter survival (**Figure 4**). A single environment, 16NY, showed intermediate survival (0.5–0.8) for its superior genotype (**Figure 4**).

Across environments, ‘Dixie’ ranked first most frequently (5 environments), followed by ‘AU Sunrise’ (4 environments), while ‘CCS Groff’, ‘Kentucky Pride’, and ‘Purple Prosperity’ most consistently appeared in the top three (9–10 environments each, **Figure 5**). The identity of the top-ranked cultivar varied considerably across environments, with no cultivar ranking first in more than five, and 13 cultivars ranking first in at least one environment, reflecting substantial genotype × environment interaction (**Figure 5**). For locations with two years of data, top-performing cultivars showed limited year-to-year consistency overall; ‘AU Sunrise’ and ‘Dixie’ in OR, ‘Dixie’ in MO and CA, ‘CCS Groff’ in WV, and ‘Purple Prosperity’ in NV were the only cultivars appearing in the top three in both years, while no overlap occurred in AR, TX, ID, or ETX (**Figure 5**).

**Figure 6:**
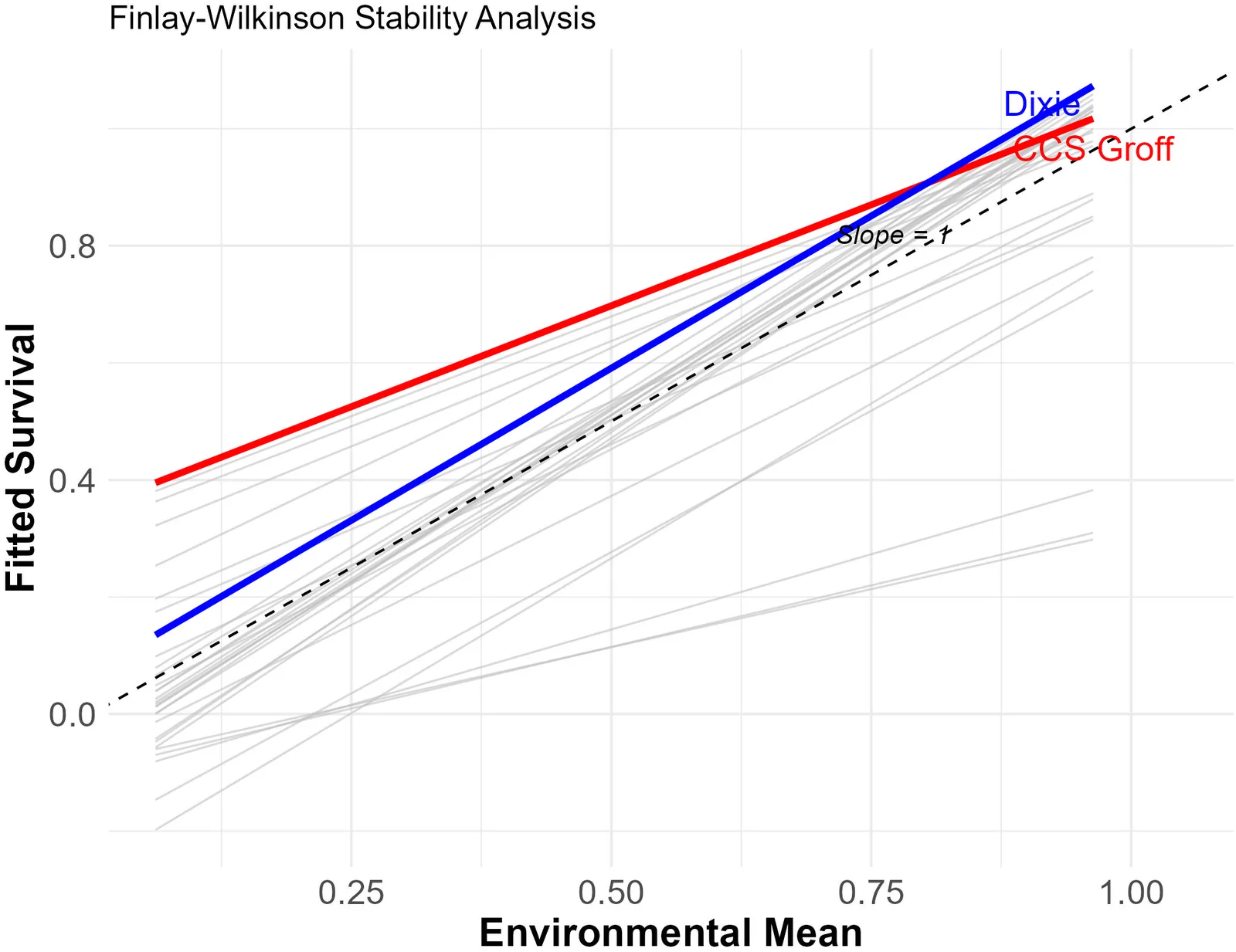
Finlay-Wilkinson stability analysis of cultivars in multi-environmental trial. Each line represents a cultivar’s regression of survival on the environmental means. A slope of 1 (dashed reference line) indicates average environmental sensitivity; slopes below 1 indicate broader stability across environments; slopes above 1 indicate greater responsiveness to environmental quality.

Finlay-Wilkinson stability analysis revealed considerable variation in both mean performance and environmental responsiveness among cultivars (**Figure 6**; **Table 5**). ‘CCS Groff’ had the highest overall mean winter survival (Mean = 0.84) combined with a slope well below 1 (b = 0.69, 95% CI: 0.48–0.90) and low stability variance (σ²d = 0.020), indicating consistently above-average performance with strong buffering under poor environments, the most desirable combination of high mean and broad stability observed in this study. ‘Purple Prosperity’ and ‘TNT’ showed similarly favorable profiles, combining high mean survival (0.81 and 0.80, respectively) with slopes below 1 (b = 0.68 for both) and low-to-moderate stability variance (σ²d = 0.032 and 0.023), indicating broad adaptability across a wide range of environments. ‘Dixie’ showed comparable overall mean survival to ‘TNT’ (mean = 0.80) but with a slope near 1 (b = 1.04, 95% CI: 0.85–1.23) and low stability variance (σ²d = 0.016), reflecting average environmental responsiveness, performing well across environments but with less buffering under poor conditions than the above cultivars. ‘Kentucky Pride’ had a similarly high overall survival (mean = 0.78) with a slope slightly above 1 (b = 1.10, 95% CI: 0.88–1.31), indicating above-average responsiveness to favorable environments but reduced stability under harsh conditions compared to ‘CCS Groff’ and ‘Purple Prosperity’. ‘Freedom’ had the lowest stability variance of all cultivars (σ²d = 0.008) with a slope significantly above 1 (b = 1.14, 95% CI: 1.01–1.27), indicating highly predictable but environmentally sensitive performance, doing well in good environments and consistently tracking the environmental trend, but not buffered against poor conditions. In contrast, ‘Dunn’ and ‘Arvica 4010’ showed the lowest mean survival (0.19 and 0.20, respectively) with slopes well below 1 (b = 0.40 and 0.42), and the highest stability variances (σ²d = 0.065 and 0.093), reflecting consistently poor and unpredictable performance regardless of environmental conditions, representing stable failure rather than stable adaptability.

**Table 5:** Mean winter survival (mean WS), Finlay-Wilkinson regression intercepts and slopes (with standard errors and 95% confidence intervals), and stability variance for each cultivar evaluated across environments in the study.

Cultivar	Mean WS	Intercept	SE intercept	Slope	SE slope	CI lower	CI upper	Stability var
CCS Groff	0.84	0.35	0.08	0.69	0.10	0.48	0.90	0.02
Purple Prosperity	0.81	0.34	0.10	0.68	0.13	0.41	0.95	0.03
TNT	0.80	0.32	0.08	0.68	0.11	0.46	0.91	0.02
Dixie	0.80	0.07	0.07	1.04	0.09	0.85	1.23	0.02
Villana	0.80	0.20	0.09	0.85	0.12	0.60	1.10	0.03
Purple Bounty	0.78	0.28	0.08	0.72	0.11	0.50	0.94	0.02
Kentucky Pride	0.78	0.01	0.08	1.10	0.10	0.88	1.31	0.02
Dynamite	0.76	-0.03	0.06	1.13	0.09	0.95	1.31	0.01
AU Sunrise	0.76	-0.07	0.08	1.18	0.11	0.96	1.40	0.02
AU Robin	0.75	-0.04	0.06	1.14	0.08	0.98	1.29	0.01
Kenland	0.74	-0.03	0.09	1.10	0.12	0.85	1.35	0.03
Cyclone II	0.74	0.00	0.07	1.06	0.10	0.86	1.25	0.02
Freedom	0.74	-0.06	0.05	1.14	0.06	1.01	1.27	0.01
Starfire II	0.74	-0.06	0.09	1.14	0.13	0.88	1.39	0.03
Fixation	0.73	-0.05	0.10	1.12	0.13	0.85	1.39	0.03
Contea	0.72	-0.05	0.08	1.11	0.10	0.90	1.32	0.02
Cinammon Plus	0.72	-0.12	0.07	1.21	0.09	1.03	1.39	0.01
AU Sunup	0.71	-0.07	0.08	1.11	0.11	0.89	1.32	0.02
Mammoth	0.71	-0.11	0.09	1.17	0.12	0.93	1.41	0.02
Wildcat	0.70	-0.13	0.08	1.19	0.10	0.98	1.40	0.02
Windham	0.69	0.15	0.11	0.77	0.15	0.45	1.08	0.04
Lana	0.65	0.13	0.15	0.75	0.20	0.35	1.15	0.07
Survivor 15	0.64	-0.01	0.08	0.92	0.11	0.69	1.15	0.02
Whistler	0.63	0.05	0.14	0.83	0.19	0.43	1.22	0.07
Lynx	0.55	-0.07	0.15	0.88	0.20	0.47	1.30	0.08
Frontier	0.48	-0.26	0.11	1.06	0.15	0.75	1.37	0.04
Frost Master	0.47	-0.21	0.13	0.97	0.17	0.61	1.32	0.05
Maxum	0.25	-0.11	0.16	0.51	0.22	0.06	0.97	0.09
Arvica 4010	0.20	-0.10	0.17	0.42	0.22	-0.04	0.88	0.09
Dunn	0.19	-0.08	0.14	0.40	0.19	0.01	0.78	0.07

### 3.2 Variance component estimation

Environmental factors accounted for the largest proportion (43.2%) of total biological variance (excluding residual), followed by GxE interaction (35.9%), genotype main effect (17.1%), and replication within environment (3.8%). Together, genotype and GxE components explained approximately 53% of the total biological variance, indicating a substantial contribution of genetic factors, both in absolute performance and in genotype-specific responses to environment, to the observed phenotypic variation. The large GxE interaction effect relative to the genotype main effect indicates that genotypic performance was considerably context-dependent, reinforcing the importance of multi-environment testing and environment-specific cultivar recommendations rather than relying on a single broadly adapted genotype. The dominant environment effect reflects the wide range of climatic conditions represented across the 30 environments included in this study.

### 3.3 Weather-trait relationships

Environment-level Pearson correlations between weather variables and winter survival were computed across 30 location-years, with Benjamini-Hochberg correction (Benjamini and Hochberg, 1994) applied across 38 simultaneous tests to control the false discovery rate. Eighteen weather variables were significantly associated with winter survival after correction (adjusted p< 0.05, **Table 6**). Temperature variables in November (TAVG.M11: r = 0.49, TMAX.M11: r = 0.47, TMIN.M11: r = 0.45), February (TAVG.M02: r = 0.44, TMAX.M02: r = 0.43, TMIN.M02: r = 0.42), March (TAVG.M03: r = 0.49, TMAX.M03: r = 0.47, TMIN.M03: r = 0.48), and October (TAVG.M10: r = 0.42, TMAX.M10: r = 0.42) showed consistent positive associations with winter survival, as did the seasonal average temperature (r = 0.43), warmer conditions across these months were associated with higher survival. In contrast, snowfall in November (r = −0.48), February (r = −0.49), March (r = −0.46), and October (r = −0.42) showed consistent negative associations, with greater snowfall associated with lower survival. Overall, November and March conditions, both temperature and snowfall, showed the most consistent associations with winter survival across environments. PC1 and PC2, which capture the dominant axes of co-variation among weather variables, were both significantly negatively associated with winter survival (r = −0.45 for each; BH-adjusted p = 0.04; **Table 6**), further supporting the pattern identified in the bivariate correlations. Given the high intercorrelations among monthly weather variables and the modest sample size (n = 30), these results should be interpreted as identifying broad seasonal windows associated with survival rather than independent causal drivers.

**Table 6:** Pearson correlations between environment-level winter survival and monthly weather variables across 30 location-years, with Benjamini-Hochberg (BH) false discovery rate correction applied across 38 simultaneous tests (n = 30 environments).

Variable	Full variable name	Correlation	P value	BH adjusted p value
Average_Temp	Overall average temperature	0.43	0.02	0.04
PC1	Principal component1	-0.45	0.01	0.04
PC2	Principal component2	-0.45	0.01	0.04
PC3	Principal component3	-0.22	0.25	0.27
PC4	Principal component4	-0.11	0.57	0.59
SNOW.M01	Snowfall in January	0.01	0.95	0.95
SNOW.M02	Snowfall in February	-0.49	0.01	0.04
SNOW.M03	Snowfall in March	-0.46	0.01	0.04
SNOW.M04	Snowfall in April	-0.40	0.03	0.06
SNOW.M09	Snowfall in September			
SNOW.M10	Snowfall in October	-0.42	0.02	0.04
SNOW.M11	Snowfall in November	-0.48	0.01	0.04
SNOW.M12	Snowfall in December	-0.20	0.28	0.30
TAVG.M01	Average temperature in January	0.28	0.13	0.15
TAVG.M02	Average temperature in February	0.44	0.01	0.04
TAVG.M03	Average temperature in March	0.49	0.01	0.04
TAVG.M04	Average temperature in April	0.36	0.05	0.08
TAVG.M09	Average temperature in September	0.39	0.03	0.06
TAVG.M10	Average temperature in October	0.42	0.02	0.04
TAVG.M11	Average temperature in November	0.49	0.01	0.04
TAVG.M12	Average temperature in December	0.35	0.06	0.08
TMAX.M01	Maximum temperature in January	0.31	0.10	0.12
TMAX.M02	Maximum temperature in February	0.43	0.02	0.04
TMAX.M03	Maximum temperature in March	0.47	0.01	0.04
TMAX.M04	Maximum temperature in April	0.35	0.06	0.08
TMAX.M09	Maximum temperature in September	0.39	0.04	0.06
TMAX.M10	Maximum temperature in October	0.42	0.02	0.04
TMAX.M11	Maximum temperature in November	0.47	0.01	0.04
TMAX.M12	Maximum temperature in December	0.33	0.07	0.09
TMIN.M01	Minimum temperature in January	0.23	0.22	0.25
TMIN.M02	Minimum temperature in February	0.42	0.02	0.04
TMIN.M03	Minimum temperature in March	0.48	0.01	0.04
TMIN.M04	Minimum temperature in April	0.34	0.06	0.09
TMIN.M09	Minimum temperature in September	0.36	0.05	0.08
TMIN.M10	Minimum temperature in October	0.37	0.04	0.07
TMIN.M11	Minimum temperature in November	0.45	0.01	0.04
TMIN.M12	Minimum temperature in December	0.32	0.08	0.10
Total_Snow	Total seasonal snowfall	-0.31	0.10	0.12

The multiple regression of environment-level survival on the first four principal components of weather variation was significant overall (F(4,25) = 3.45, p = 0.022, adjusted R² = 0.25), indicating that weather variation collectively explained approximately 25% of the variance in environment-level survival. Variance inflation factors were low (range 1.00–1.16), indicating negligible multicollinearity among the components. However, no individual principal component reached conventional significance (PC1: p = 0.070; PC2: p = 0.065; PC3: p = 0.182; PC4: p = 0.559), suggesting that survival responded to a broad pattern of co-varying climatic conditions rather than any single dominant weather axis.

## 4 Discussion

Winter survival is a critical determinant of winter cover crop performance. Low winter survival reduces biomass production, limits root development that stabilizes soil, and negatively impacts soil erosion control, water infiltration, and nitrogen fixation (Florence et al., 2019; Kaye et al., 2019; Ranaldo et al., 2020). Winter legume cover crop species vary in winter hardiness, with hairy vetch generally considered the most hardy; however, substantial variability exists among genotypes. In this study, 30 commercial cultivars of winter annual legumes, hairy vetch, crimson clover, red clover, balansa clover, and winter pea, were evaluated for winter survival across 30 (after filtering) unique environments.

Variance partitioning indicated that environmental factors accounted for the largest proportion of variance in winter survival (43.2%), followed by G×E interaction (35.9%) and genotype main effect (17.1%). The substantial G×E component, comparable in magnitude to the environmental effect and larger than the genotype main effect, indicates that cultivar performance was often context-dependent, with many cultivars re-ranking meaningfully across environments rather than shifting uniformly. This finding reinforces the value of multi-environment testing and cautions against generalizing single-environment recommendations.

Several methods, including AMMI, GGE biplots, and hierarchical clustering, were explored to group environments, but clear distinctions were not achieved. Winter survival is a complex trait influenced by the interplay of multiple climatic, edaphic, and biological factors, no single one of which dominates consistently across environments (Zheng et al., 2018; Loel and Hoffmann, 2014), making it inherently difficult to group environments based on genotype response patterns.

Year-to-year variation in winter survival within the same locations was substantial and represents one of the most practically important findings of this study. For example, winter survival in 17WA (<0.1) and 17WV (0.19) were far lower than in 16WA (0.72) and 16WV (0.85), while 17ID (0.85) exceeded 16ID (0.64) (**Table 7**). This degree of interannual variability underscores the risks of relying on single-year evaluations for cultivar recommendations, as a cultivar identified as superior in one year may perform poorly in the next due to climatic variation alone. With increasing climatic unpredictability, multi-year, multi-environment testing becomes increasingly critical for generating reliable recommendations for growers.

**Table 7:** Average winter survival for each environment in multi-environmental trials.

S. N	Environment	Average survival
1	16AR	0.79
2	17AR	0.72
3	16AZ	0.64
4	17AZ	1.00
5	16CA	0.85
6	17CA	0.94
7	17CO	0.19
8	16ETX	0.63
9	17ETX	0.84
10	17GA	0.86
11	16ID	0.64
12	17ID	0.85
13	16KS	0.81
14	16MD	0.69
15	16MI	0.66
16	16MO	0.53
17	17MO	0.46
18	16MS	0.96
19	17MS	0.82
20	16NM	0.89
21	16NV	0.48
22	17NV	0.55
23	16NY	0.30
24	16OR	0.71
25	17OR	0.78
26	16TX	0.72
27	17TX	0.89
28	16WA	0.72
29	17WA	0.08
30	16WV	0.85
31	17WV	0.19

17AZ was removed from the analysis.

Correlation analyses suggested that winter survival was associated with a broad pattern of co-varying temperature and snowfall conditions across months rather than any single dominant climatic factor, consistent with the complexity of winter survival as a trait. The negative association between snowfall and winter survival observed here may appear counterintuitive, as snow cover is generally expected to insulate plants from extreme freezing temperatures (Loel and Hoffmann, 2014). However, most environments in this study were comparatively mild, as reflected by generally high winter survival across cultivars. In such environments, snow accumulation may reduce survival through at least two mechanisms rather than providing a net protective effect. First, snow cover creates sustained cold, humid conditions at the plant canopy that favor psychrophilic fungal pathogens, particularly *Sclerotinia trifoliorum*, which causes clover rot in legumes including red clover O’Sullivan et al., 2021). This pathogen’s interaction with freezing stress has been shown to negatively affect winter survival in red clover, with combined freezing and infection having a greater negative effect than either stress alone (Ergon and Amdahl, 2024). Several other fungal pathogens, such as *Microdochium nivale* (pink snow mold), also remain active above -5 °C (Hoshino et al., 2009), and snow mold damage has been directly observed in crimson clover under snow cover at mild temperatures (Graybill, 2023). Second, snowmelt and prolonged soil moisture associated with snow cover may cause waterlogging and associated hypoxic or anoxic conditions in the root zone, to which legumes such as hairy vetch and peas are sensitive (Malik et al., 2015; Wiraguna et al., 2021); waterlogging has been shown to reduce photosynthesis, halt root development, and reduce seed production in peas (Ploschuk et al., 2018). Together, these pathogen and waterlogging effects suggest that in mild environments, the costs of snow cover can outweigh its insulating benefits. This aligns with the idea that snow’s protective role may depend on climate severity: in more extreme cold environments, prior studies suggest snow cover can buffer plants from the most severe freezing damage (Leep et al., 1992; Sheaffer, 2023). At the environment level, snowfall in this dataset may also act as a proxy for colder, harsher winter conditions more broadly, as locations receiving more snowfall tended to experience lower temperatures overall, making it difficult to fully separate the direct effects of snow cover from the broader climatic severity of those environments.

Additional unmeasured factors beyond temperature and snowfall, such as freezing duration (Zheng et al., 2018), freezing without snow cover (Loel and Hoffmann, 2014), soil characteristics including depth and texture (Kucek et al., 2024), and ecological and edaphic variability (Boukrouh et al., 2023), may have further contributed to survival outcomes in some environments. The frequency of freeze-thaw cycles, minimum freezing temperature, and pre-existing cold acclimation and local adaptation can also influence site and cultivar specific response to freezing (Dietrich et al., 2018). Management practices such as planting date, weed management, and irrigation likely contributed as well. Although planting dates followed regional recommendations at each site, these recommendations differ across locations, and combined with differential irrigation, plants could have grown differently and entered winter at different developmental stages with different degrees of cold acclimation (Lim et al., 2014; Thapa et al., 2025). This is a plausible source of the environment and G×E effects observed, since insufficiently hardened or acclimated stands are more vulnerable to winterkill. Because these practices varied across sites, their individual effects could not be isolated here and represent an important direction for future work.

Among the cultivars evaluated, not all cultivars were equally sensitive to environment. Hairy vetch cultivars (‘CCS Groff’, ‘Purple Prosperity’, and ‘TNT’) combined high overall winter survival with slopes below 1, indicating broad adaptability and strong buffering under poor environments; the most desirable combination for general recommendation. However, this conclusion should be interpreted cautiously given that only 30 environments across two years were evaluated and the cultivar set was limited; these cultivars demonstrated relatively broad adaptation within the environments tested rather than universal adaptability. Notably, the exclusion of environment 17AZ, where mean winter survival reached 100% with zero variance across all genotypes, serves as an important reminder that even the poorest-performing cultivars in this study possess the physiological capacity for complete survival under sufficiently favorable conditions, suggesting that the performance differences observed here reflect responses to environmental stress rather than absolute limitations in survival potential.

Locations 17CO and 17WA, exhibited less than 0.5 winter survival even for the best performing genotypes (**Figure 4**). The low survival observed in harsh winter environments underscores the need to develop/identify more hardy and stable genotypes for such US environments. Potential strategies include targeted germplasm exploration of northern-adapted and winter-hardy ecotypes, phenotypic screening under controlled freezing conditions to identify tolerance mechanisms, incorporation of locally adapted wild relatives or landrace material, and application of genomic selection approaches that could accelerate identification of cold-hardiness alleles across species and breeding populations.

This study was conducted in 2016–2018, and many newer cultivars have been released after the study period. Whether these newer cultivars offer improved stability or performance in colder environments remains to be determined. Furthermore, only one (two in TX) location per state were tested, despite substantial within-state variation, so each state’s result reflects a single site’s soil and microclimate and should be extrapolated within states with caution. Incorporating more locations within each state and conducting more regional trials could improve the identification of cultivars that are stable and well-adapted in a region. Additionally, the study spans only two years; given the substantial interannual variability observed across locations, conclusions about cultivar stability and broad adaptability should be interpreted with this temporal limitation in mind. Another limitation involves the relatively high proportion of plots with spring counts exceeding fall counts (16.6%), which likely reflects biological processes inherent to cover crop systems, including germination of dormant seeds after fall counting, spring tillering that inflated visual plant counts, and the difficulty of accurately counting dense canopies in field conditions. While the sensitivity analysis confirmed that capping these values did not materially affect genotypic predictions, this rate highlights the challenge of using plant counts as a proxy for winter survival in cover crops and suggests that future studies might benefit from alternative assessment methods, such as percent ground cover or biomass estimates, which may be less susceptible to this confounding. Finally, this article focuses solely on winter survival. Although winter survival is a strong predictor of biomass and overall cover crop performance, the cultivar rankings here should be interpreted as survival-specific rather than as overall performance rankings, and evaluating additional traits, such as vigor, biomass, emergence, and nitrogen fixation in future studies can provide more comprehensive assessment of cultivar performance. .

**Figure 7:**
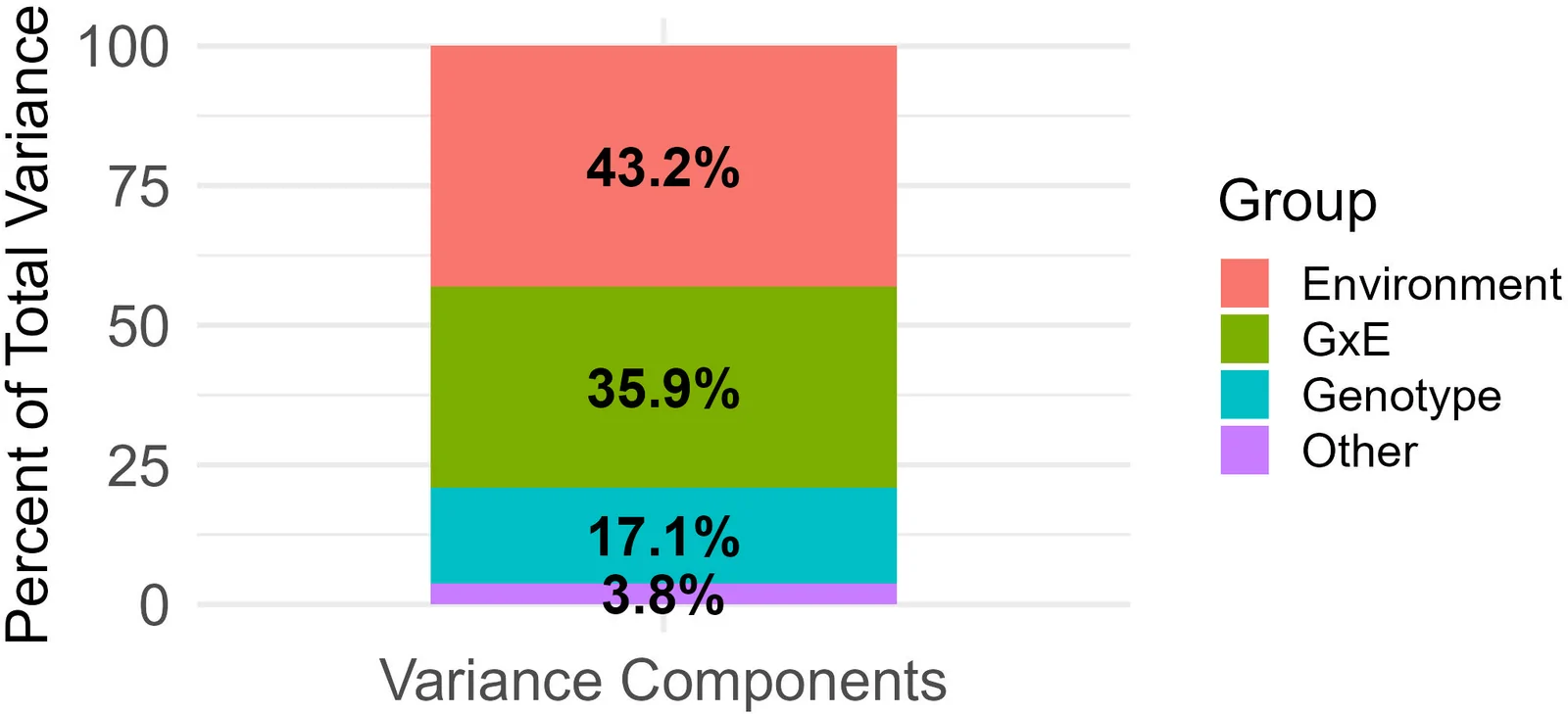
Partitioning of the total variance in winter survival into its component sources. Bars show the percentage of total variance attributable to environment, genotype × environment interaction (G×E), genotype, and replicate within environment (Other).

## 5 Conclusion

This study evaluated 30 legume cover crop cultivars across 31 environments to identify superior and stable genotypes, determine key weather variables influencing performance, and provide location recommendations. Environment and G×E interaction were the dominant sources of variation, exceeding the genotype main effect, indicating that cultivar performance was considerably context-dependent and that environment-specific recommendations are warranted. Among the cultivars evaluated, hairy vetch cultivars ‘CCS Groff’, ‘Purple Prosperity’, and ‘TNT’ demonstrated the highest overall survival combined with strong buffering under poor environments and the broadest adaptability within the range of environments tested. Crimson clover cultivars ‘Dixie’ and ‘Kentucky Pride’ also performed well but showed greater responsiveness to favorable environments and less stability under harsh conditions. The substantial interannual variability observed across locations underscores the importance of multi-year, multi-environment testing for reliable cultivar recommendations. Temperatures in November, February, and March (positive effect) and snowfall in these same months (negative effect) were most consistently associated with winter survival, though no single climatic factor dominated, reflecting the complexity of the trait. Environments such as 17WA and 17CO exhibited less than 0.5 survival even for top-performing cultivars, highlighting the need to identify or develop more cold-hardy genotypes through targeted germplasm exploration, winter hardiness screening, and genomic selection approaches. This need is likely to grow as winter climates become more variable and freeze–thaw events more frequent. However, winter survival alone is not a sufficient basis for cultivar recommendation, as a plant may survive winter but emerge with poor spring vigor or reduced biomass, limiting its practical value as a cover crop. Future studies should assess additional agronomic traits such as spring vigor, biomass, and nitrogen fixation, incorporate newly released cultivars, and expand geographic and temporal coverage to provide a more comprehensive basis for cultivar recommendations.

## Data Availability

The raw data supporting the conclusions of this article will be made available by the authors, without undue reservation.

## References

[B2] Benjamini Y. Hochberg Y. (1994). Controlling the false discovery rate: A practical and powerful approach to multiple testing. R. Stat. Soc 57, 289–300. doi: 10.1111/j.2517-6161.1995.tb02031.x 40046247

[B4] Boukrouh S. Noutfia A. Moula N. Avril C. Louvieaux J. Hornick J. L. . (2023). Ecological, morpho-agronomical, and bromatological assessment of sorghum ecotypes in Northern Morocco. Sci. Rep. 13, 1–16. doi: 10.1038/s41598-023-41565-9 37730680 PMC10511723

[B3] Boukrouh S. Noutfia A. Moula N. Avril C. Louvieaux J. Hornick J. . (2024). Characterisation of bitter vetch (Vicia ervilia (L.) Willd) ecotypes: An ancient and promising legume. 60, 1–18. doi: 10.1017/S0014479724000139

[B5] Butler D. G. Cullis B. R. Gilmour A. R. Gogel B. J. Thompson R. Biology C. (2023). ASReml-R reference manual. Available online at: https://asreml.kb.vsni.co.uk/wp-content/uploads/sites/3/ASReml-R-Reference-Manual-4.2.pdf.

[B6] Casey A. Spellman D. (2020). Evaluation of annual cool season cover crop varieties for the inland pacific northwest. Available online at: https://www.nrcs.usda.gov/plantmaterials/wapmcsr13617.pdf.

[B7] Dietrich C. C. Kreyling J. Jentsch A. Malyshev A. V. (2018). Intraspecific variation in response to magnitude and frequency of freeze-thaw cycles in a temperate grass 1–13. doi: 10.1093/aobpla/plx068 PMC5751040 29308126

[B8] Drescher M. Thomas S. C. (2013). Snow cover manipulations alter survival of early life stages of cold-temperate tree species. Oikos 122, 541–554. doi: 10.1111/j.1600-0706.2012.20642.x 40046247

[B9] Ergon Å. Amdahl H. (2024). Winter survival in red clover: Experimental evidence for interactions among stresses. BMC Plant Biol. 24, 1–13. doi: 10.1186/s12870-024-05167-5 38807057 PMC11131274

[B501] Finlay K. W. Wilkinson G. N. (1963). The analysis of adaptation in a plant-breeding programme. Aust. J. Agric. Res. 14, 742–754. doi: 10.1071/AR9630742

[B10] Florence A. M. Higley L. G. Drijber R. A. Francis C. A. Lindquist J. L. (2019). Cover crop mixture diversity, biomass productivity, weed suppression, and stability. PloS One 14, 1–18. doi: 10.1371/journal.pone.0206195 30870424 PMC6417710

[B11] Graybill J. (2023). “ Winter survival of small grains and cover crops at risk,” in Pennstate Ext. Available online at: https://extension.psu.edu/winter-survival-of-small-grains-and-cover-crops-at-risk

[B12] Hadasch S. Forkman J. Malik W. A. Piepho H. P. (2018). Weighted estimation of AMMI and GGE models. J. Agric. Biol. Environ. Stat. 23, 255–275. doi: 10.1007/s13253-018-0323-z 30311153

[B13] Herget M. (2020). Evaluation of selected cool season cover crop varieties in northeast missouri. Available online at: https://www.midwestcovercrops.org/wp-content/uploads/2021/05/MO_2020_Evaluation-of-Cool-Season-Cover-Crop-Varieties.pdf

[B14] Hoshino T. Xiao N. Tkachenko O. B. (2009). Cold adaptation in the phytopathogenic fungi causing snow molds. 50, 26–38. doi: 10.1007/s10267-008-0452-2

[B15] Kaye J. Finney D. White C. Bradley B. Schipanski M. Alonso-Ayuso M. . (2019). Managing nitrogen through cover crop species selection in the U.S. Mid-Atlantic. PloS One 14, 1–23. doi: 10.1371/journal.pone.0215448 30978240 PMC6461281

[B16] Kucek L. K. Moore V. M. Muller K. Reberg-horton C. Martins L. B. Mirsky S. B. . (2024). Genetic and environmental drivers of legume cover crop performance: Hairy vetch. 64, 3052–3072. doi: 10.1002/csc2.21318

[B17] Leep R. H. Andresen J. A. Jeranyama P. (1992). Fall dormancy and snow depth effects on winterkill of alfalfa. 93, 1142–1148. doi: 10.2134/agronj2001.9351142x

[B18] Lim C. K. Krebs S. L. Arora R. (2014). Cold hardiness increases with age in juvenile Rhododendron populations. 5, 1–7. doi: 10.3389/fpls.2014.00542 PMC4199316 25360138

[B19] Loel J. Hoffmann C. M. (2014). Importance of growth stage and weather conditions for the winter hardiness of autumn sown sugar beet. F. Crop Res. 162, 70–76. doi: 10.1016/j.fcr.2014.03.007 38826717

[B20] Malik A. I. Ailewe T. I. Erskine W. (2015). Special issue: Plant responses to low-oxygen environments tolerance of three grain legume species to transient waterlogging. 7, 1–11. doi: 10.1093/aobpla/plv040 PMC4455828 25902834

[B21] Monge-Sanz B. (2024). Global warming may be behind an increase in the frequency and intensity of cold spellse. PreventionWeb. doi: 10.64628/ab.avg4pq9gj

[B22] Moore V. M. Maul J. E. Wilson D. Curran W. S. Brainard D. C. Devine T. E. . (2020). Registration of ‘Purple Bounty’ and ‘Purple Prosperity’ hairy vetch. J. Plant Regist. 14, 340–346. doi: 10.1002/plr2.20044 41531421

[B23] Okii D. Mukankusi C. Sebuliba S. Tukamuhabwa P. Tusiime G. Talwana H. . (2018). Genetic variation, heritability estimates and GXE effects on yield traits of Mesoamerican common bean (Phaseolus vulgaris L) germplasm in Uganda. Plant Genet. Resour. Char. Util. 16, 237–248. doi: 10.1017/S1479262117000259 41292463

[B24] Piepho H. Mohring J. Melchinger A. (2008). BLUP for phenotypic selection in plant breeding and variety testing. Euphytica 161, 209–228. doi: 10.1007/s10681-007-9449-8 30311153

[B25] Ploschuk R. A. Miralles D. J. Colmer T. D. Ploschuk E. L. Striker G. G. (2018). Waterlogging of winter crops at early and late stages: Impacts on leaf physiology, growth and yield. 9, 1–15. doi: 10.3389/fpls.2018.01863 PMC6306497 30619425

[B26] PRISM Climate Group (2024). AN91m Dataset, 4 Km Spatial Resolution (Corvallis, OR: Oregon State University). Available online at: https://prism.oregonstate.edu (Accessed September, 2024).

[B27] Qian B. Jing Q. Jégo G. Bélanger G. Smith W. Vanderzaag A. (2024). Warmer future climate in Canada — implications for winter survival of perennial forage crops. 335, 320–335. doi: 10.1139/cjps-2023-0192

[B28] Ranaldo M. Carlesi S. Costanzo A. Bàrberi P. (2020). Functional diversity of cover crop mixtures enhances biomass yield and weed suppression in a Mediterranean agroecosystem. Weed. Res. 60, 96–108. doi: 10.1111/wre.12388 40046247

[B29] SARE-CTIC (2016). Annual Report 2015–2016 Cover Crop Survey (College Park, MD; West Lafayette: Sustainable Agriculture Research & Education-Conservation Technology Information Center). Available online at: https://www.sare.org/wp-content/uploads/2015-2016-Cover-Crop-Survey-Report.pdf (Accessed February 2, 2026).

[B30] SARE-CTIC (2017). Annual Report 2016–2017 Cover Crop Survey (College Park, MD; West Lafayette: Sustainable Agriculture Research & Education-Conservation Technology Information Center). Available online at: https://www.sare.org/wp-content/uploads/2016-2017-Cover-Crop-Survey-Report.pdf (Accessed February 2, 2026).

[B31] SARE-CTIC (2020). Annual Report 2019–2020 National Cover Crop Survey (College Park, MD; West Lafayette: Sustainable Agriculture Research & Education-Conservation Technology Information Center). Available online at: https://www.sare.org/wp-content/uploads/2019-2020-National-Cover-Crop-Survey.pdf (Accessed February 2, 2026).

[B32] SARE-CTIC (2023). National Cover Crop Survey Report 2022–2023 (College Park, MD; West Lafayette: Sustainable Agriculture Research & Education-Conservation Technology Information Center). Available online at: https://www.ctic.org/files/2022-2023%20National%20Cover%20Crop%20Survey%20Report-%20FNL%281%29.pdf (Accessed February 2, 2026).

[B33] Secchi M. A. Bastos L. M. Stamm M. J. Wright Y. Foster C. Messina C. D. . (2021). Winter survival response of canola to meteorological variables and adaptative areas for current canola germplasm in the United States. Agric. For. Meteorol. 297, 108267. doi: 10.1016/j.agrformet.2020.108267 38826717

[B34] Sheaffer C. (2023). “ Let it snow!,” in Univ. Minnesota Ext. Available online at: https://blog-crop-news.extension.umn.edu/2023/01/let-it-snow.html

[B35] Sullivan C. A. O. Belt K. Thatcher L. F. Jimenez-gasco M. D. M. Thatcher L. F. (2021). Tackling control of a cosmopolitan phytopathogen: Sclerotinia. 12, 1–18. doi: 10.3389/fpls.2021.707509 PMC8417578 34490008

[B36] Thapa R. Hanson S. Hua J. Moore V. M. (2025). Breeding for cold tolerance in common annual legume cover crops. Crop Sci. 65, 1–18. doi: 10.1002/csc2.70059 41531421

[B37] Thomas J. B. Schaalje G. B. Grant M. N. (1993). Survival, height and genotype by environment interaction in winter wheat. Can. J. Plant Sci. 73, 417–427. doi: 10.4141/cjps93-061

[B38] Thurston C. L. Grossman J. M. Fudge R. Maul J. E. Mirsky S. Wiering N. (2022). Cold stress reduces nodulation and symbiotic nitrogen fixation in winter annual legume cover crops. Plant Soil 481, 661–676. doi: 10.1007/s11104-022-05667-z 30311153

[B39] USDA-NASS (2024). 2022 Census of Agriculture (Washington, DC: United States Department of Agriculture, National Agricultural Statistics Service). Available online at: https://www.nass.usda.gov/AgCensus/ (Accessed February 2, 2026).

[B40] Vann R. A. Reberg-Horton S. C. Castillo M. S. Murphy J. P. Martins L. B. Mirsky S. B. . (2021). Differences among eighteen winter pea genotypes for forage and cover crop use in the southeastern United States. Crop Sci. 61, 947–965. doi: 10.1002/csc2.20355 41531421

[B41] Wiraguna E. Malik A. I. Colmer T. D. Erskine W. (2021). Tolerance of four grain legume species to waterlogging, hypoxia and anoxia at germination and recovery. 13, 1–13. doi: 10.1093/aobpla/plab052 PMC8405847 34476049

[B42] Zheng D. Yang X. Mínguez M. I. Mu C. He Q. Wu X. (2018). Effect of freezing temperature and duration on winter survival and grain yield of winter wheat. Agric. For. Meteorol. 260–261, 1–8. doi: 10.1016/j.agrformet.2018.05.011 38826717

