# Supplementary material for: Genotype by environment interaction and local adaptation of commercial legume cover crop genotypes
Source: Front Plant Sci. 2026 Aug 10;17:1903778. doi: 10.3389/fpls.2026.1903778 (PMC13500589; doi:10.3389/fpls.2026.1903778)
Supplement: Supplementary file 1 [file DataSheet1.docx]

Supplementary Material

# 1. Supplementary methods

## Environment cluster analysis:

Environmental clustering was initially attempted using the Additive Main Effect and Multiplicative Interactions (AMMI) and Genotype plus Genotype by Environment Interaction (GGE) models. However, distinct and biologically meaningful clusters were not identified. Principal components derived from the GGE model were subsequently used to determine the optimal number of clusters through Silhouette width, elbow method, and gap statistics. These approaches yielded inconsistent results, and neither the gap statistic nor the elbow method provided distinct optimal number of clusters. Hierarchical clustering was also explored using the number of clusters suggested by these methods, but the resulting groupings lacked interpretability and biological relevance. Due to the absence of coherent and reproducible clusters, environmental cluster analysis was ultimately excluded from the study.

Supplementary Figure 1. Sample plot plan for each environment

# 2. Supplementary Data

Supplementary Table 1. Mean and standard deviation of survival for each environment

| LocYear | Mean survival | Standar deviation of winter survival | Number of cultivars | Number of observations |
| --- | --- | --- | --- | --- |
| 17AZ | 1 | 0 | 26 | 102 |
| 16MS | 0.96 | 0.07 | 16 | 61 |
| 16WV | 0.84 | 0.14 | 11 | 44 |
| 17CA | 0.94 | 0.15 | 23 | 88 |
| 17GA | 0.87 | 0.18 | 24 | 72 |
| 16AZ | 0.64 | 0.19 | 30 | 119 |
| 17WA | 0.08 | 0.20 | 29 | 114 |
| 16CA | 0.84 | 0.20 | 29 | 114 |
| 17ETX | 0.83 | 0.22 | 29 | 111 |
| 16NY | 0.30 | 0.23 | 19 | 74 |
| 16WA | 0.72 | 0.24 | 29 | 115 |
| 17CO | 0.19 | 0.26 | 19 | 68 |
| 16KS | 0.81 | 0.26 | 26 | 102 |
| 16NM | 0.88 | 0.27 | 28 | 112 |
| 17TX | 0.89 | 0.29 | 29 | 112 |
| 16ETX | 0.63 | 0.31 | 28 | 111 |
| 17ID | 0.85 | 0.31 | 30 | 120 |
| 17MS | 0.82 | 0.33 | 25 | 91 |
| 16TX | 0.71 | 0.34 | 28 | 110 |
| 17AR | 0.73 | 0.34 | 27 | 107 |
| 17WV | 0.19 | 0.34 | 27 | 107 |
| 16MO | 0.53 | 0.34 | 25 | 94 |
| 17OR | 0.78 | 0.35 | 30 | 118 |
| 17NV | 0.52 | 0.36 | 30 | 112 |
| 16OR | 0.71 | 0.36 | 30 | 118 |
| 16MD | 0.69 | 0.37 | 29 | 113 |
| 16AR | 0.78 | 0.37 | 30 | 120 |
| 16NV | 0.49 | 0.38 | 22 | 81 |
| 16ID | 0.64 | 0.39 | 30 | 120 |
| 17MO | 0.50 | 0.39 | 18 | 68 |
| 16MI | 0.64 | 0.43 | 30 | 116 |

Supplementary Table 2. Best Linear Unbiased Predictions (BLUPs) of winter survival from logistic regression model used in the study.

| LocYear | Cultivar | Predicted value | Std error | Transformed value | Transformed Value std error |
| --- | --- | --- | --- | --- | --- |
| 16NM | Dixie | 7.96 | 1.83 | 1.00 | 0.00 |
| 16NM | Kentucky Pride | 7.91 | 1.84 | 1.00 | 0.00 |
| 16NM | AU Robin | 7.31 | 1.83 | 1.00 | 0.00 |
| 16NM | Fixation | 7.31 | 1.83 | 1.00 | 0.00 |
| 16CA | Dixie | 7.07 | 1.33 | 1.00 | 0.00 |
| 16AR | Dynamite | 7.06 | 1.80 | 1.00 | 0.00 |
| 16AR | Kenland | 6.99 | 1.80 | 1.00 | 0.00 |
| 16AR | Cyclone II | 6.97 | 1.76 | 1.00 | 0.00 |
| 16AR | Freedom | 6.96 | 1.78 | 1.00 | 0.00 |
| 16ID | Kenland | 6.86 | 1.15 | 1.00 | 0.00 |
| 16AR | Villana | 6.60 | 2.03 | 1.00 | 0.01 |
| 16CA | Kentucky Pride | 6.54 | 1.43 | 1.00 | 0.00 |
| 16AR | Starfire II | 6.48 | 1.91 | 1.00 | 0.01 |
| 16AR | Wildcat | 6.42 | 1.84 | 1.00 | 0.01 |
| 17TX | CCS Groff | 6.42 | 1.49 | 1.00 | 0.01 |
| 17TX | Kentucky Pride | 6.40 | 1.39 | 1.00 | 0.00 |
| 17TX | AU Sunrise | 6.35 | 1.32 | 1.00 | 0.00 |
| 17ETX | Freedom | 6.30 | 1.37 | 1.00 | 0.01 |
| 17TX | Purple Prosperity | 6.30 | 1.39 | 1.00 | 0.01 |
| 17TX | TNT | 6.29 | 1.28 | 1.00 | 0.00 |
| 17CA | Dixie | 6.29 | 1.65 | 1.00 | 0.01 |
| 17TX | Dixie | 6.27 | 1.49 | 1.00 | 0.01 |
| 17ETX | Kentucky Pride | 6.26 | 1.57 | 1.00 | 0.01 |
| 17TX | Villana | 6.17 | 1.50 | 1.00 | 0.01 |
| 17CA | AU Robin | 6.16 | 1.42 | 1.00 | 0.01 |
| 17CA | Purple Prosperity | 6.14 | 1.67 | 1.00 | 0.01 |
| 17CA | Villana | 6.13 | 1.69 | 1.00 | 0.01 |
| 16MI | Kentucky Pride | 6.11 | 1.72 | 1.00 | 0.01 |
| 17ETX | Mammoth | 6.09 | 1.38 | 1.00 | 0.01 |
| 17CA | Purple Bounty | 6.03 | 1.55 | 1.00 | 0.01 |
| 17CA | TNT | 6.02 | 1.64 | 1.00 | 0.01 |
| 16MI | Wildcat | 5.99 | 1.54 | 1.00 | 0.01 |
| 17CA | Starfire II | 5.96 | 1.49 | 1.00 | 0.01 |
| 17ETX | AU Robin | 5.92 | 1.49 | 1.00 | 0.01 |
| 16MI | AU Robin | 5.90 | 1.63 | 1.00 | 0.01 |
| 17TX | AU Robin | 5.89 | 1.31 | 1.00 | 0.01 |
| 17TX | Freedom | 5.87 | 1.24 | 1.00 | 0.01 |
| 17TX | Fixation | 5.84 | 1.36 | 1.00 | 0.01 |
| 17TX | Purple Bounty | 5.83 | 1.50 | 1.00 | 0.01 |
| 17ETX | Dynamite | 5.80 | 1.52 | 1.00 | 0.01 |
| 17ETX | Cyclone II | 5.76 | 1.46 | 1.00 | 0.01 |
| 17MS | Cyclone II | 5.67 | 1.21 | 1.00 | 0.01 |
| 17TX | Contea | 5.65 | 1.35 | 1.00 | 0.01 |
| 17CA | AU Sunrise | 5.62 | 2.04 | 1.00 | 0.02 |
| 17TX | Starfire II | 5.59 | 1.49 | 1.00 | 0.01 |
| 17TX | Kenland | 5.59 | 1.49 | 1.00 | 0.01 |
| 17MS | Starfire II | 5.58 | 1.28 | 1.00 | 0.01 |
| 17TX | Mammoth | 5.54 | 1.33 | 1.00 | 0.01 |
| 17TX | Cinammon Plus | 5.51 | 1.34 | 1.00 | 0.01 |
| 17OR | AU Sunrise | 5.50 | 1.22 | 1.00 | 0.01 |
| 17CA | Whistler | 5.47 | 1.34 | 1.00 | 0.01 |
| 16NM | CCS Groff | 5.45 | 3.54 | 1.00 | 0.12 |
| 17MS | AU Sunrise | 5.45 | 1.47 | 1.00 | 0.01 |
| 17MS | Dixie | 5.44 | 1.53 | 1.00 | 0.02 |
| 17TX | Windham | 5.42 | 1.29 | 1.00 | 0.01 |
| 17MS | Wildcat | 5.41 | 1.24 | 1.00 | 0.01 |
| 17CA | Dynamite | 5.41 | 1.81 | 1.00 | 0.02 |
| 17ETX | Starfire II | 5.39 | 1.63 | 1.00 | 0.02 |
| 17MS | Cinammon Plus | 5.36 | 1.27 | 1.00 | 0.01 |
| 17OR | Dixie | 5.34 | 1.28 | 1.00 | 0.01 |
| 17TX | AU Sunup | 5.28 | 1.51 | 0.99 | 0.02 |
| 17CA | Lana | 5.25 | 1.49 | 0.99 | 0.02 |
| 16NM | Dynamite | 5.24 | 0.78 | 0.99 | 0.01 |
| 17MS | Kenland | 5.23 | 1.37 | 0.99 | 0.02 |
| 16NM | Villana | 5.20 | 3.55 | 0.99 | 0.15 |
| 17TX | Lynx | 5.20 | 1.05 | 0.99 | 0.01 |
| 17TX | Wildcat | 5.20 | 1.49 | 0.99 | 0.02 |
| 16NM | Purple Prosperity | 5.20 | 3.55 | 0.99 | 0.16 |
| 17CA | Kenland | 5.18 | 1.96 | 0.99 | 0.03 |
| 17MS | Dynamite | 5.17 | 1.41 | 0.99 | 0.02 |
| 16NV | Purple Prosperity | 5.15 | 1.78 | 0.99 | 0.03 |
| 17TX | Whistler | 5.14 | 1.21 | 0.99 | 0.01 |
| 16NM | AU Sunrise | 5.13 | 3.55 | 0.99 | 0.17 |
| 17CA | Fixation | 5.10 | 2.09 | 0.99 | 0.04 |
| 16NM | Cyclone II | 5.06 | 0.54 | 0.99 | 0.00 |
| 16KS | Purple Prosperity | 5.05 | 1.04 | 0.99 | 0.01 |
| 17TX | Survivor 15 | 5.05 | 1.19 | 0.99 | 0.01 |
| 16NM | TNT | 5.02 | 3.54 | 0.99 | 0.18 |
| 17CA | Freedom | 4.99 | 2.03 | 0.99 | 0.04 |
| 17MS | AU Robin | 4.99 | 1.46 | 0.99 | 0.02 |
| 17TX | Lana | 4.88 | 1.49 | 0.99 | 0.02 |
| 16NM | Purple Bounty | 4.86 | 3.55 | 0.99 | 0.20 |
| 17MS | AU Sunup | 4.86 | 1.40 | 0.99 | 0.02 |
| 16NM | Maxum | 4.86 | 1.47 | 0.99 | 0.03 |
| 17CA | Cinammon Plus | 4.83 | 2.03 | 0.99 | 0.05 |
| 17CA | Mammoth | 4.82 | 2.04 | 0.99 | 0.05 |
| 17CA | AU Sunup | 4.81 | 2.04 | 0.99 | 0.05 |
| 17CA | Frost Master | 4.81 | 1.30 | 0.99 | 0.02 |
| 17GA | Dixie | 4.81 | 1.09 | 0.99 | 0.02 |
| 17MS | Mammoth | 4.80 | 1.42 | 0.99 | 0.02 |
| 17ID | Windham | 4.79 | 0.96 | 0.99 | 0.01 |
| 17OR | Purple Bounty | 4.75 | 1.31 | 0.99 | 0.02 |
| 16TX | Lana | 4.74 | 0.93 | 0.99 | 0.01 |
| 17ETX | CCS Groff | 4.72 | 2.59 | 0.99 | 0.10 |
| 16NM | Wildcat | 4.69 | 0.61 | 0.99 | 0.01 |
| 16KS | Villana | 4.64 | 1.14 | 0.99 | 0.02 |
| 16NM | Kenland | 4.62 | 3.54 | 0.99 | 0.24 |
| 17OR | AU Sunup | 4.62 | 1.23 | 0.99 | 0.02 |
| 17GA | Kentucky Pride | 4.59 | 1.15 | 0.99 | 0.02 |
| 17ETX | Dixie | 4.57 | 2.59 | 0.99 | 0.11 |
| 17ID | Purple Prosperity | 4.54 | 0.85 | 0.99 | 0.01 |
| 17MS | Kentucky Pride | 4.53 | 2.13 | 0.99 | 0.07 |
| 17ID | CCS Groff | 4.52 | 0.88 | 0.99 | 0.01 |
| 17OR | Kentucky Pride | 4.51 | 0.64 | 0.99 | 0.01 |
| 16NM | Freedom | 4.51 | 3.54 | 0.99 | 0.27 |
| 16MD | Kentucky Pride | 4.45 | 0.90 | 0.99 | 0.02 |
| 17ID | TNT | 4.44 | 0.84 | 0.99 | 0.02 |
| 17CA | CCS Groff | 4.43 | 1.17 | 0.99 | 0.03 |
| 17ETX | AU Sunrise | 4.39 | 2.60 | 0.99 | 0.13 |
| 16KS | Dynamite | 4.38 | 1.08 | 0.99 | 0.02 |
| 16ID | Dynamite | 4.33 | 0.40 | 0.99 | 0.01 |
| 16NM | Mammoth | 4.33 | 3.55 | 0.99 | 0.30 |
| 16ID | Wildcat | 4.33 | 0.43 | 0.99 | 0.01 |
| 16NM | AU Sunup | 4.32 | 3.55 | 0.99 | 0.30 |
| 17MS | Contea | 4.29 | 1.70 | 0.99 | 0.06 |
| 16AR | Contea | 4.28 | 0.60 | 0.99 | 0.01 |
| 16NM | Arvica 4010 | 4.26 | 0.65 | 0.99 | 0.01 |
| 17CA | Wildcat | 4.26 | 1.03 | 0.99 | 0.02 |
| 16CA | CCS Groff | 4.24 | 2.67 | 0.99 | 0.16 |
| 17ID | Mammoth | 4.23 | 0.80 | 0.99 | 0.02 |
| 16MS | Freedom | 4.23 | 0.85 | 0.99 | 0.02 |
| 17ID | Lynx | 4.21 | 0.93 | 0.99 | 0.02 |
| 16NM | Cinammon Plus | 4.21 | 0.53 | 0.99 | 0.01 |
| 16AR | CCS Groff | 4.21 | 3.92 | 0.99 | 0.41 |
| 16CA | Whistler | 4.20 | 0.74 | 0.99 | 0.02 |
| 16MI | Mammoth | 4.20 | 0.76 | 0.99 | 0.02 |
| 17TX | Cyclone II | 4.18 | 0.83 | 0.98 | 0.02 |
| 17TX | Dynamite | 4.17 | 0.87 | 0.98 | 0.02 |
| 16NM | Starfire II | 4.16 | 0.55 | 0.98 | 0.01 |
| 16NM | Windham | 4.14 | 3.55 | 0.98 | 0.34 |
| 16OR | AU Sunrise | 4.14 | 0.46 | 0.98 | 0.01 |
| 17ID | Wildcat | 4.10 | 0.57 | 0.98 | 0.01 |
| 17GA | AU Sunup | 4.10 | 1.04 | 0.98 | 0.03 |
| 16AR | Dixie | 4.05 | 3.92 | 0.98 | 0.45 |
| 17ETX | Cinammon Plus | 4.05 | 0.53 | 0.98 | 0.01 |
| 16AR | Kentucky Pride | 4.04 | 3.93 | 0.98 | 0.45 |
| 17ID | Kentucky Pride | 4.04 | 1.62 | 0.98 | 0.06 |
| 17ETX | Fixation | 4.03 | 1.00 | 0.98 | 0.03 |
| 17AR | TNT | 4.02 | 0.81 | 0.98 | 0.02 |
| 17ID | Kenland | 4.02 | 0.52 | 0.98 | 0.01 |
| 16ID | Cyclone II | 4.01 | 0.36 | 0.98 | 0.01 |
| 16ID | Cinammon Plus | 4.00 | 0.42 | 0.98 | 0.01 |
| 16ID | Starfire II | 3.98 | 0.43 | 0.98 | 0.01 |
| 17ID | Villana | 3.95 | 1.63 | 0.98 | 0.07 |
| 16CA | AU Sunrise | 3.91 | 2.67 | 0.98 | 0.21 |
| 16KS | Kenland | 3.89 | 1.19 | 0.98 | 0.04 |
| 17ETX | Kenland | 3.88 | 2.59 | 0.98 | 0.20 |
| 17ID | AU Sunrise | 3.88 | 1.63 | 0.98 | 0.07 |
| 17ID | Dynamite | 3.87 | 0.63 | 0.98 | 0.02 |
| 16KS | CCS Groff | 3.86 | 1.63 | 0.98 | 0.08 |
| 17GA | CCS Groff | 3.86 | 1.57 | 0.98 | 0.07 |
| 17MS | Fixation | 3.85 | 2.18 | 0.98 | 0.14 |
| 16KS | AU Robin | 3.83 | 0.58 | 0.98 | 0.02 |
| 16KS | Starfire II | 3.81 | 1.22 | 0.98 | 0.05 |
| 16OR | Purple Prosperity | 3.80 | 0.82 | 0.98 | 0.03 |
| 17AR | CCS Groff | 3.80 | 0.92 | 0.98 | 0.03 |
| 17ID | Dixie | 3.79 | 0.66 | 0.98 | 0.02 |
| 16KS | Contea | 3.78 | 0.83 | 0.98 | 0.03 |
| 16AR | TNT | 3.77 | 3.92 | 0.98 | 0.51 |
| 16AR | Purple Prosperity | 3.76 | 0.67 | 0.98 | 0.02 |
| 17ETX | Contea | 3.75 | 2.60 | 0.98 | 0.22 |
| 17MS | Freedom | 3.75 | 2.12 | 0.98 | 0.14 |
| 17TX | Frost Master | 3.73 | 0.92 | 0.98 | 0.03 |
| 17AR | Purple Prosperity | 3.73 | 0.88 | 0.98 | 0.03 |
| 17OR | Wildcat | 3.71 | 0.72 | 0.98 | 0.02 |
| 16MD | Purple Bounty | 3.71 | 0.95 | 0.98 | 0.04 |
| 16MS | AU Sunrise | 3.66 | 1.26 | 0.97 | 0.06 |
| 16KS | Dixie | 3.66 | 0.74 | 0.97 | 0.03 |
| 17AR | Villana | 3.64 | 0.91 | 0.97 | 0.04 |
| 16AR | Purple Bounty | 3.61 | 3.92 | 0.97 | 0.55 |
| 17ID | Purple Bounty | 3.61 | 1.62 | 0.97 | 0.09 |
| 17GA | Villana | 3.61 | 1.58 | 0.97 | 0.09 |
| 16AR | AU Sunrise | 3.60 | 0.61 | 0.97 | 0.02 |
| 17ID | Cyclone II | 3.59 | 0.58 | 0.97 | 0.02 |
| 17ETX | AU Sunup | 3.58 | 2.60 | 0.97 | 0.25 |
| 16AR | AU Sunup | 3.58 | 0.60 | 0.97 | 0.02 |
| 16MI | CCS Groff | 3.57 | 3.39 | 0.97 | 0.43 |
| 16TX | Purple Prosperity | 3.56 | 0.47 | 0.97 | 0.02 |
| 16MS | TNT | 3.56 | 1.24 | 0.97 | 0.06 |
| 16MS | Dixie | 3.54 | 0.90 | 0.97 | 0.04 |
| 17GA | AU Sunrise | 3.53 | 1.58 | 0.97 | 0.10 |
| 16MS | AU Robin | 3.53 | 0.61 | 0.97 | 0.02 |
| 16OR | Dixie | 3.50 | 0.50 | 0.97 | 0.02 |
| 16OR | Kentucky Pride | 3.49 | 0.50 | 0.97 | 0.02 |
| 16MS | Starfire II | 3.48 | 0.51 | 0.97 | 0.02 |
| 17TX | Frontier | 3.47 | 1.56 | 0.97 | 0.10 |
| 17AR | Purple Bounty | 3.46 | 0.88 | 0.97 | 0.04 |
| 16CA | AU Robin | 3.45 | 2.66 | 0.97 | 0.28 |
| 16TX | Villana | 3.44 | 0.64 | 0.97 | 0.03 |
| 16KS | TNT | 3.43 | 1.62 | 0.97 | 0.11 |
| 17CA | Cyclone II | 3.42 | 0.65 | 0.97 | 0.03 |
| 16MI | Dixie | 3.42 | 3.39 | 0.97 | 0.46 |
| 17ETX | Windham | 3.40 | 2.60 | 0.97 | 0.28 |
| 16MS | Purple Bounty | 3.40 | 1.25 | 0.97 | 0.07 |
| 16CA | Fixation | 3.39 | 2.71 | 0.97 | 0.30 |
| 16MS | Purple Prosperity | 3.38 | 0.92 | 0.97 | 0.05 |
| 16MS | Fixation | 3.37 | 0.84 | 0.97 | 0.04 |
| 16AR | Fixation | 3.36 | 3.95 | 0.97 | 0.61 |
| 17ID | Fixation | 3.36 | 1.69 | 0.97 | 0.12 |
| 16MI | Villana | 3.32 | 3.40 | 0.97 | 0.48 |
| 16MI | Purple Prosperity | 3.31 | 3.39 | 0.96 | 0.49 |
| 17GA | Purple Prosperity | 3.30 | 0.98 | 0.96 | 0.05 |
| 16MS | Cinammon Plus | 3.30 | 0.67 | 0.96 | 0.03 |
| 17ID | AU Robin | 3.29 | 0.60 | 0.96 | 0.03 |
| 16CA | Freedom | 3.29 | 2.67 | 0.96 | 0.31 |
| 16MS | Villana | 3.28 | 0.95 | 0.96 | 0.05 |
| 16KS | Purple Bounty | 3.27 | 1.63 | 0.96 | 0.13 |
| 16CA | Contea | 3.27 | 2.67 | 0.96 | 0.32 |
| 16MS | Windham | 3.27 | 1.00 | 0.96 | 0.06 |
| 16MD | CCS Groff | 3.25 | 0.83 | 0.96 | 0.04 |
| 16MI | AU Sunrise | 3.24 | 3.40 | 0.96 | 0.50 |
| 16MI | AU Sunup | 3.24 | 0.61 | 0.96 | 0.03 |
| 16MS | Dynamite | 3.22 | 1.24 | 0.96 | 0.08 |
| 16MD | Purple Prosperity | 3.17 | 0.81 | 0.96 | 0.05 |
| 16CA | Cyclone II | 3.16 | 2.67 | 0.96 | 0.34 |
| 17MS | TNT | 3.16 | 0.82 | 0.96 | 0.05 |
| 16MS | Kenland | 3.15 | 1.25 | 0.96 | 0.09 |
| 17ID | Cinammon Plus | 3.14 | 0.56 | 0.96 | 0.03 |
| 16MI | TNT | 3.14 | 3.39 | 0.96 | 0.52 |
| 16CA | Cinammon Plus | 3.12 | 2.67 | 0.96 | 0.35 |
| 17ID | Freedom | 3.12 | 0.45 | 0.96 | 0.02 |
| 16CA | AU Sunup | 3.10 | 2.68 | 0.96 | 0.35 |
| 16MD | Contea | 3.10 | 0.77 | 0.96 | 0.05 |
| 16ID | Mammoth | 3.10 | 0.38 | 0.96 | 0.02 |
| 16TX | CCS Groff | 3.10 | 1.86 | 0.96 | 0.18 |
| 16AR | Cinammon Plus | 3.09 | 3.92 | 0.96 | 0.65 |
| 16AR | Mammoth | 3.08 | 3.93 | 0.96 | 0.66 |
| 17GA | AU Robin | 3.07 | 1.56 | 0.96 | 0.14 |
| 17ID | AU Sunup | 3.07 | 1.63 | 0.96 | 0.15 |
| 17CA | Survivor 15 | 3.05 | 0.47 | 0.95 | 0.03 |
| 17OR | Villana | 3.03 | 2.30 | 0.95 | 0.28 |
| 16KS | Fixation | 3.02 | 1.70 | 0.95 | 0.16 |
| 16MS | Contea | 3.02 | 0.86 | 0.95 | 0.06 |
| 17AR | Kentucky Pride | 3.01 | 0.52 | 0.95 | 0.03 |
| 16ETX | AU Sunrise | 3.01 | 0.41 | 0.95 | 0.02 |
| 16TX | Whistler | 3.00 | 0.58 | 0.95 | 0.03 |
| 16MD | Villana | 3.00 | 0.83 | 0.95 | 0.06 |
| 17MS | CCS Groff | 3.00 | 0.85 | 0.95 | 0.06 |
| 17OR | Starfire II | 3.00 | 0.60 | 0.95 | 0.04 |
| 17CA | Frontier | 2.99 | 2.08 | 0.95 | 0.24 |
| 16MI | Purple Bounty | 2.98 | 3.39 | 0.95 | 0.55 |
| 17ETX | Whistler | 2.98 | 2.59 | 0.95 | 0.36 |
| 16CA | Arvica 4010 | 2.97 | 0.63 | 0.95 | 0.04 |
| 16KS | Kentucky Pride | 2.94 | 0.57 | 0.95 | 0.03 |
| 17GA | Dynamite | 2.94 | 0.77 | 0.95 | 0.05 |
| 16TX | AU Robin | 2.93 | 0.49 | 0.95 | 0.03 |
| 16OR | AU Sunup | 2.92 | 0.41 | 0.95 | 0.02 |
| 16CA | Windham | 2.92 | 2.68 | 0.95 | 0.39 |
| 16TX | Dixie | 2.92 | 0.41 | 0.95 | 0.02 |
| 17GA | Lynx | 2.92 | 0.71 | 0.95 | 0.05 |
| 16NM | Contea | 2.92 | 0.52 | 0.95 | 0.03 |
| 16CA | TNT | 2.91 | 0.48 | 0.95 | 0.03 |
| 17GA | Contea | 2.89 | 1.58 | 0.95 | 0.16 |
| 16TX | Lynx | 2.87 | 0.57 | 0.95 | 0.04 |
| 16MS | Mammoth | 2.87 | 1.26 | 0.95 | 0.11 |
| 16OR | Contea | 2.84 | 0.37 | 0.95 | 0.02 |
| 16OR | CCS Groff | 2.83 | 1.21 | 0.94 | 0.11 |
| 16NV | Whistler | 2.83 | 0.48 | 0.94 | 0.03 |
| 17ETX | Survivor 15 | 2.82 | 2.60 | 0.94 | 0.39 |
| 16MI | Dynamite | 2.80 | 3.39 | 0.94 | 0.59 |
| 17GA | Cyclone II | 2.78 | 1.57 | 0.94 | 0.17 |
| 17ID | Starfire II | 2.78 | 0.44 | 0.94 | 0.03 |
| 16TX | AU Sunrise | 2.77 | 1.87 | 0.94 | 0.23 |
| 17GA | Fixation | 2.76 | 0.79 | 0.94 | 0.06 |
| 16KS | Cinammon Plus | 2.75 | 1.62 | 0.94 | 0.18 |
| 17GA | Cinammon Plus | 2.75 | 1.57 | 0.94 | 0.18 |
| 16KS | Mammoth | 2.74 | 1.64 | 0.94 | 0.19 |
| 16MI | Starfire II | 2.74 | 3.39 | 0.94 | 0.60 |
| 17GA | Mammoth | 2.74 | 1.58 | 0.94 | 0.18 |
| 16KS | AU Sunup | 2.73 | 1.64 | 0.94 | 0.19 |
| 16MI | Fixation | 2.72 | 3.43 | 0.94 | 0.61 |
| 17ETX | Wildcat | 2.71 | 0.52 | 0.94 | 0.04 |
| 16MS | Lana | 2.71 | 0.81 | 0.94 | 0.07 |
| 17CA | Kentucky Pride | 2.68 | 0.55 | 0.94 | 0.04 |
| 16TX | Contea | 2.67 | 0.41 | 0.94 | 0.03 |
| 16AR | Lana | 2.67 | 3.92 | 0.94 | 0.71 |
| 17AR | Dixie | 2.65 | 1.29 | 0.93 | 0.14 |
| 17CA | Contea | 2.64 | 0.55 | 0.93 | 0.04 |
| 16TX | TNT | 2.62 | 0.48 | 0.93 | 0.04 |
| 16MS | AU Sunup | 2.62 | 0.74 | 0.93 | 0.06 |
| 16MI | Freedom | 2.62 | 3.39 | 0.93 | 0.62 |
| 17CA | Windham | 2.60 | 0.61 | 0.93 | 0.05 |
| 16MI | Contea | 2.60 | 3.40 | 0.93 | 0.62 |
| 17GA | Whistler | 2.60 | 0.74 | 0.93 | 0.07 |
| 16MS | Survivor 15 | 2.59 | 0.79 | 0.93 | 0.07 |
| 16MS | Cyclone II | 2.58 | 0.53 | 0.93 | 0.04 |
| 17MS | Villana | 2.58 | 0.57 | 0.93 | 0.05 |
| 16AR | Windham | 2.57 | 0.50 | 0.93 | 0.04 |
| 16CA | Lana | 2.57 | 0.55 | 0.93 | 0.05 |
| 17ETX | Lynx | 2.57 | 2.59 | 0.93 | 0.44 |
| 17ID | Contea | 2.57 | 0.52 | 0.93 | 0.04 |
| 16KS | AU Sunrise | 2.56 | 0.48 | 0.93 | 0.04 |
| 17AR | Fixation | 2.56 | 1.08 | 0.93 | 0.11 |
| 17MS | Lynx | 2.55 | 2.12 | 0.93 | 0.32 |
| 17GA | Kenland | 2.54 | 0.64 | 0.93 | 0.06 |
| 17GA | Windham | 2.54 | 1.59 | 0.93 | 0.20 |
| 16WV | AU Sunrise | 2.52 | 0.61 | 0.93 | 0.05 |
| 16KS | Cyclone II | 2.51 | 0.54 | 0.92 | 0.05 |
| 17OR | Dynamite | 2.51 | 2.29 | 0.92 | 0.37 |
| 16NM | Frontier | 2.51 | 3.57 | 0.92 | 0.67 |
| 16WV | CCS Groff | 2.50 | 0.60 | 0.92 | 0.05 |
| 17MS | Purple Bounty | 2.49 | 0.65 | 0.92 | 0.06 |
| 16WA | Cyclone II | 2.49 | 0.38 | 0.92 | 0.03 |
| 17OR | AU Robin | 2.49 | 2.29 | 0.92 | 0.37 |
| 17AR | AU Sunrise | 2.47 | 1.31 | 0.92 | 0.16 |
| 16MO | CCS Groff | 2.47 | 0.60 | 0.92 | 0.06 |
| 16CA | Mammoth | 2.47 | 0.40 | 0.92 | 0.03 |
| 16MI | Cinammon Plus | 2.46 | 3.39 | 0.92 | 0.64 |
| 17OR | Kenland | 2.44 | 2.29 | 0.92 | 0.38 |
| 16TX | Frost Master | 2.44 | 0.42 | 0.92 | 0.04 |
| 16MS | Frost Master | 2.44 | 0.65 | 0.92 | 0.06 |
| 17OR | Lana | 2.43 | 0.45 | 0.92 | 0.04 |
| 17OR | Fixation | 2.43 | 2.35 | 0.92 | 0.40 |
| 16MS | Kentucky Pride | 2.42 | 0.49 | 0.92 | 0.05 |
| 17OR | Freedom | 2.40 | 0.41 | 0.92 | 0.04 |
| 17ID | Whistler | 2.40 | 0.48 | 0.92 | 0.05 |
| 17OR | TNT | 2.39 | 0.52 | 0.92 | 0.05 |
| 16MI | Cyclone II | 2.39 | 0.42 | 0.92 | 0.04 |
| 17OR | Purple Prosperity | 2.37 | 0.58 | 0.91 | 0.06 |
| 16OR | Villana | 2.36 | 0.53 | 0.91 | 0.05 |
| 16CA | Survivor 15 | 2.34 | 2.67 | 0.91 | 0.49 |
| 16KS | Lana | 2.33 | 1.63 | 0.91 | 0.24 |
| 16TX | Dynamite | 2.33 | 1.86 | 0.91 | 0.30 |
| 16MD | Lana | 2.32 | 0.62 | 0.91 | 0.06 |
| 16AR | Survivor 15 | 2.31 | 3.93 | 0.91 | 0.74 |
| 17OR | Contea | 2.31 | 2.30 | 0.91 | 0.41 |
| 17ID | Survivor 15 | 2.31 | 1.63 | 0.91 | 0.25 |
| 16ID | Dixie | 2.30 | 2.84 | 0.91 | 0.54 |
| 17MS | Purple Prosperity | 2.30 | 0.74 | 0.91 | 0.08 |
| 16ID | Kentucky Pride | 2.29 | 2.85 | 0.91 | 0.54 |
| 17GA | Frost Master | 2.28 | 0.73 | 0.91 | 0.08 |
| 16CA | Lynx | 2.27 | 0.59 | 0.91 | 0.06 |
| 16TX | Kenland | 2.26 | 1.86 | 0.91 | 0.31 |
| 16TX | Fixation | 2.25 | 1.92 | 0.90 | 0.32 |
| 16OR | AU Robin | 2.25 | 0.38 | 0.90 | 0.04 |
| 16MI | Windham | 2.25 | 3.40 | 0.90 | 0.66 |
| 17CA | Dunn | 2.25 | 0.45 | 0.90 | 0.05 |
| 16MS | Whistler | 2.24 | 1.24 | 0.90 | 0.17 |
| 16WA | Windham | 2.24 | 0.49 | 0.90 | 0.05 |
| 17NV | Cyclone II | 2.22 | 0.39 | 0.90 | 0.04 |
| 17MS | Lana | 2.22 | 0.50 | 0.90 | 0.05 |
| 17OR | Cyclone II | 2.20 | 2.29 | 0.90 | 0.42 |
| 16ID | Villana | 2.20 | 2.85 | 0.90 | 0.56 |
| 16MD | AU Sunrise | 2.18 | 1.57 | 0.90 | 0.25 |
| 16AZ | Kenland | 2.18 | 0.55 | 0.90 | 0.06 |
| 16WA | Wildcat | 2.17 | 0.38 | 0.90 | 0.04 |
| 17OR | Cinammon Plus | 2.16 | 2.29 | 0.90 | 0.43 |
| 16MS | Wildcat | 2.16 | 0.41 | 0.90 | 0.04 |
| 17OR | Mammoth | 2.16 | 2.30 | 0.90 | 0.43 |
| 17GA | Starfire II | 2.15 | 0.61 | 0.90 | 0.07 |
| 16TX | Freedom | 2.15 | 1.86 | 0.90 | 0.32 |
| 17MS | Frost Master | 2.14 | 2.13 | 0.89 | 0.39 |
| 16KS | Whistler | 2.12 | 1.62 | 0.89 | 0.27 |
| 16CA | Maxum | 2.10 | 0.44 | 0.89 | 0.05 |
| 16ID | Purple Prosperity | 2.09 | 0.42 | 0.89 | 0.05 |
| 16TX | Purple Bounty | 2.09 | 0.41 | 0.89 | 0.05 |
| 16OR | TNT | 2.08 | 0.49 | 0.89 | 0.06 |
| 17WV | Purple Prosperity | 2.08 | 0.45 | 0.89 | 0.05 |
| 16MD | TNT | 2.08 | 1.55 | 0.89 | 0.26 |
| 16TX | Survivor 15 | 2.07 | 0.43 | 0.89 | 0.05 |
| 16WA | Dynamite | 2.07 | 0.44 | 0.89 | 0.05 |
| 16OR | Purple Bounty | 2.07 | 0.53 | 0.89 | 0.06 |
| 16AR | Lynx | 2.05 | 3.92 | 0.89 | 0.75 |
| 16WA | AU Robin | 2.05 | 0.39 | 0.89 | 0.05 |
| 16OR | Cinammon Plus | 2.05 | 0.37 | 0.89 | 0.04 |
| 16ID | TNT | 2.02 | 2.84 | 0.88 | 0.58 |
| 17AR | AU Robin | 2.01 | 1.29 | 0.88 | 0.21 |
| 17AR | Freedom | 2.01 | 0.38 | 0.88 | 0.05 |
| 17MO | Dixie | 2.00 | 0.61 | 0.88 | 0.08 |
| 16OR | Starfire II | 2.00 | 1.21 | 0.88 | 0.19 |
| 16MD | Starfire II | 2.00 | 0.56 | 0.88 | 0.07 |
| 16OR | Fixation | 1.99 | 1.31 | 0.88 | 0.22 |
| 16TX | Cinammon Plus | 1.98 | 1.86 | 0.88 | 0.35 |
| 16WA | Dixie | 1.98 | 0.37 | 0.88 | 0.04 |
| 17CA | Maxum | 1.98 | 0.60 | 0.88 | 0.08 |
| 16WV | Kentucky Pride | 1.98 | 0.90 | 0.88 | 0.13 |
| 17GA | Freedom | 1.97 | 0.47 | 0.88 | 0.06 |
| 17AR | Starfire II | 1.97 | 1.29 | 0.88 | 0.21 |
| 16TX | AU Sunup | 1.96 | 1.87 | 0.88 | 0.35 |
| 17CA | Arvica 4010 | 1.95 | 0.47 | 0.88 | 0.06 |
| 16KS | Wildcat | 1.94 | 0.47 | 0.87 | 0.06 |
| 16KS | Freedom | 1.94 | 0.49 | 0.87 | 0.06 |
| 16NV | Maxum | 1.90 | 0.48 | 0.87 | 0.07 |
| 16TX | Kentucky Pride | 1.90 | 0.40 | 0.87 | 0.05 |
| 16NV | Lana | 1.90 | 0.51 | 0.87 | 0.07 |
| 16WA | AU Sunrise | 1.90 | 0.39 | 0.87 | 0.05 |
| 16WV | Villana | 1.89 | 0.90 | 0.87 | 0.14 |
| 17GA | Survivor 15 | 1.88 | 0.70 | 0.87 | 0.10 |
| 17AR | Dynamite | 1.86 | 0.41 | 0.87 | 0.06 |
| 16ID | Purple Bounty | 1.86 | 2.84 | 0.87 | 0.59 |
| 16AZ | Starfire II | 1.86 | 0.54 | 0.86 | 0.08 |
| 17GA | Wildcat | 1.84 | 0.53 | 0.86 | 0.08 |
| 17MO | Starfire II | 1.84 | 0.83 | 0.86 | 0.13 |
| 16MS | Lynx | 1.83 | 1.25 | 0.86 | 0.22 |
| 17AR | Contea | 1.83 | 1.30 | 0.86 | 0.23 |
| 16MI | Whistler | 1.83 | 3.39 | 0.86 | 0.69 |
| 16WA | Kentucky Pride | 1.79 | 0.38 | 0.86 | 0.05 |
| 16OR | Dynamite | 1.78 | 0.37 | 0.86 | 0.05 |
| 17NV | Dynamite | 1.78 | 0.41 | 0.86 | 0.06 |
| 16TX | Windham | 1.78 | 1.87 | 0.86 | 0.38 |
| 17CA | Lynx | 1.77 | 0.54 | 0.85 | 0.08 |
| 17MS | Frontier | 1.75 | 2.17 | 0.85 | 0.46 |
| 17GA | Frontier | 1.73 | 1.23 | 0.85 | 0.23 |
| 16MD | AU Robin | 1.72 | 1.55 | 0.85 | 0.30 |
| 16WV | TNT | 1.71 | 0.88 | 0.85 | 0.15 |
| 16WV | Purple Prosperity | 1.71 | 0.49 | 0.85 | 0.08 |
| 16WA | Starfire II | 1.70 | 1.63 | 0.85 | 0.33 |
| 16WA | Kenland | 1.70 | 1.63 | 0.85 | 0.33 |
| 16ID | CCS Groff | 1.69 | 0.45 | 0.84 | 0.07 |
| 16WA | Fixation | 1.69 | 1.71 | 0.84 | 0.35 |
| 16CA | Villana | 1.68 | 0.41 | 0.84 | 0.06 |
| 16MI | Survivor 15 | 1.67 | 3.40 | 0.84 | 0.69 |
| 16MD | Fixation | 1.67 | 1.63 | 0.84 | 0.33 |
| 16ID | AU Robin | 1.66 | 2.84 | 0.84 | 0.60 |
| 16MS | CCS Groff | 1.65 | 0.65 | 0.84 | 0.11 |
| 16ETX | Lana | 1.65 | 0.38 | 0.84 | 0.06 |
| 17ID | Frost Master | 1.64 | 1.62 | 0.84 | 0.33 |
| 17NV | Purple Prosperity | 1.64 | 0.50 | 0.84 | 0.08 |
| 17NV | Starfire II | 1.64 | 0.52 | 0.84 | 0.08 |
| 16WA | Cinammon Plus | 1.63 | 0.36 | 0.84 | 0.06 |
| 17WV | TNT | 1.63 | 0.42 | 0.84 | 0.07 |
| 16WA | Lynx | 1.62 | 0.51 | 0.84 | 0.08 |
| 16CA | Frost Master | 1.62 | 0.46 | 0.84 | 0.07 |
| 16WV | AU Robin | 1.62 | 0.49 | 0.83 | 0.08 |
| 16OR | Wildcat | 1.61 | 1.20 | 0.83 | 0.23 |
| 16MI | Kenland | 1.61 | 0.40 | 0.83 | 0.06 |
| 16NV | Arvica 4010 | 1.61 | 0.56 | 0.83 | 0.09 |
| 16ID | Fixation | 1.61 | 2.88 | 0.83 | 0.62 |
| 17OR | CCS Groff | 1.60 | 0.47 | 0.83 | 0.08 |
| 17MO | Kentucky Pride | 1.59 | 0.71 | 0.83 | 0.12 |
| 17AR | Wildcat | 1.58 | 1.29 | 0.83 | 0.26 |
| 16WA | Freedom | 1.57 | 0.39 | 0.83 | 0.06 |
| 17MO | CCS Groff | 1.57 | 1.12 | 0.83 | 0.22 |
| 16WA | Contea | 1.56 | 1.64 | 0.83 | 0.35 |
| 16MD | Freedom | 1.56 | 1.55 | 0.83 | 0.32 |
| 16WV | Purple Bounty | 1.55 | 0.89 | 0.83 | 0.17 |
| 16ETX | CCS Groff | 1.54 | 0.36 | 0.82 | 0.06 |
| 17GA | Lana | 1.52 | 0.48 | 0.82 | 0.08 |
| 16OR | Freedom | 1.52 | 0.35 | 0.82 | 0.06 |
| 16OR | Windham | 1.51 | 1.23 | 0.82 | 0.25 |
| 16OR | Mammoth | 1.51 | 0.36 | 0.82 | 0.06 |
| 16WV | Dixie | 1.51 | 0.51 | 0.82 | 0.09 |
| 17WV | CCS Groff | 1.50 | 0.42 | 0.82 | 0.07 |
| 16ID | Freedom | 1.50 | 2.84 | 0.82 | 0.61 |
| 16AZ | Villana | 1.50 | 1.85 | 0.82 | 0.40 |
| 17AR | Cyclone II | 1.49 | 0.37 | 0.82 | 0.06 |
| 16AZ | Purple Prosperity | 1.49 | 1.84 | 0.82 | 0.40 |
| 16CA | Wildcat | 1.49 | 0.38 | 0.82 | 0.06 |
| 16MD | Kenland | 1.47 | 0.49 | 0.81 | 0.09 |
| 16MD | Whistler | 1.46 | 0.57 | 0.81 | 0.10 |
| 16MD | Cyclone II | 1.43 | 1.55 | 0.81 | 0.34 |
| 17AR | Mammoth | 1.43 | 0.38 | 0.81 | 0.07 |
| 17NV | Dixie | 1.42 | 1.53 | 0.81 | 0.33 |
| 16MI | Lynx | 1.42 | 3.39 | 0.80 | 0.68 |
| 16WA | Mammoth | 1.41 | 1.64 | 0.80 | 0.36 |
| 17ETX | Frontier | 1.40 | 0.61 | 0.80 | 0.11 |
| 16MD | Cinammon Plus | 1.40 | 1.55 | 0.80 | 0.34 |
| 16AZ | Windham | 1.39 | 0.46 | 0.80 | 0.08 |
| 16MD | Mammoth | 1.39 | 1.57 | 0.80 | 0.34 |
| 16OR | Kenland | 1.39 | 0.35 | 0.80 | 0.06 |
| 17WV | Purple Bounty | 1.38 | 0.37 | 0.80 | 0.07 |
| 17OR | Survivor 15 | 1.38 | 2.30 | 0.80 | 0.51 |
| 16MD | AU Sunup | 1.38 | 1.57 | 0.80 | 0.35 |
| 16WV | Dynamite | 1.37 | 0.87 | 0.80 | 0.18 |
| 16MD | Dixie | 1.37 | 0.43 | 0.80 | 0.08 |
| 17MO | TNT | 1.37 | 0.40 | 0.80 | 0.07 |
| 17MO | Villana | 1.36 | 0.46 | 0.80 | 0.08 |
| 16CA | Dunn | 1.35 | 0.47 | 0.79 | 0.09 |
| 17MO | Wildcat | 1.35 | 0.66 | 0.79 | 0.13 |
| 17MO | Mammoth | 1.35 | 0.85 | 0.79 | 0.17 |
| 16MO | TNT | 1.34 | 0.50 | 0.79 | 0.09 |
| 16ETX | Windham | 1.34 | 0.37 | 0.79 | 0.07 |
| 16MO | Dixie | 1.33 | 0.91 | 0.79 | 0.19 |
| 17NV | Villana | 1.32 | 1.54 | 0.79 | 0.34 |
| 16MO | Kentucky Pride | 1.31 | 0.92 | 0.79 | 0.19 |
| 16WV | Starfire II | 1.31 | 0.88 | 0.79 | 0.18 |
| 16CA | Kenland | 1.31 | 0.41 | 0.79 | 0.08 |
| 16WV | Kenland | 1.31 | 0.88 | 0.79 | 0.18 |
| 16KS | Frost Master | 1.30 | 1.63 | 0.79 | 0.37 |
| 16WV | Fixation | 1.30 | 1.01 | 0.79 | 0.21 |
| 16OR | Lana | 1.30 | 1.21 | 0.79 | 0.26 |
| 17OR | Windham | 1.30 | 0.45 | 0.79 | 0.09 |
| 16CA | Frontier | 1.29 | 2.71 | 0.78 | 0.59 |
| 17GA | TNT | 1.28 | 0.57 | 0.78 | 0.11 |
| 16AR | AU Robin | 1.28 | 0.36 | 0.78 | 0.07 |
| 17NV | AU Robin | 1.27 | 0.45 | 0.78 | 0.09 |
| 17AR | Lana | 1.27 | 1.29 | 0.78 | 0.29 |
| 16AR | Frontier | 1.26 | 3.95 | 0.78 | 0.71 |
| 17ID | Frontier | 1.25 | 1.68 | 0.78 | 0.38 |
| 16MO | Dynamite | 1.25 | 0.45 | 0.78 | 0.09 |
| 17AR | Cinammon Plus | 1.25 | 0.40 | 0.78 | 0.08 |
| 16WA | Frost Master | 1.23 | 0.43 | 0.77 | 0.08 |
| 16MO | Villana | 1.23 | 0.92 | 0.77 | 0.20 |
| 16MO | Purple Prosperity | 1.22 | 0.91 | 0.77 | 0.20 |
| 16OR | Cyclone II | 1.20 | 0.35 | 0.77 | 0.07 |
| 16CA | Purple Prosperity | 1.20 | 0.44 | 0.77 | 0.09 |
| 16WV | Freedom | 1.19 | 0.88 | 0.77 | 0.19 |
| 17AR | Kenland | 1.17 | 0.43 | 0.76 | 0.09 |
| 16ID | AU Sunrise | 1.15 | 0.35 | 0.76 | 0.07 |
| 16MO | AU Sunrise | 1.15 | 0.93 | 0.76 | 0.20 |
| 16AZ | Lana | 1.13 | 0.39 | 0.76 | 0.08 |
| 16MD | Survivor 15 | 1.13 | 0.44 | 0.76 | 0.09 |
| 17MO | Kenland | 1.13 | 0.63 | 0.76 | 0.13 |
| 16ETX | Whistler | 1.12 | 0.36 | 0.75 | 0.07 |
| 16NV | TNT | 1.10 | 0.49 | 0.75 | 0.10 |
| 16MO | Purple Bounty | 1.10 | 0.51 | 0.75 | 0.11 |
| 16ETX | Dixie | 1.10 | 1.54 | 0.75 | 0.36 |
| 17ETX | Villana | 1.09 | 0.51 | 0.75 | 0.11 |
| 16NV | Villana | 1.09 | 0.50 | 0.75 | 0.11 |
| 16ETX | Kentucky Pride | 1.09 | 1.56 | 0.75 | 0.36 |
| 16WV | Contea | 1.08 | 0.52 | 0.75 | 0.11 |
| 16WV | Cyclone II | 1.07 | 0.88 | 0.74 | 0.20 |
| 17NV | CCS Groff | 1.07 | 0.53 | 0.74 | 0.11 |
| 17MO | Cinammon Plus | 1.07 | 0.54 | 0.74 | 0.12 |
| 16NM | Whistler | 1.05 | 0.54 | 0.74 | 0.12 |
| 16NY | Villana | 1.04 | 0.53 | 0.74 | 0.12 |
| 16MS | Frontier | 1.04 | 1.33 | 0.74 | 0.31 |
| 16WV | Cinammon Plus | 1.03 | 0.87 | 0.74 | 0.20 |
| 16WV | Mammoth | 1.02 | 0.90 | 0.74 | 0.20 |
| 16AZ | Whistler | 1.02 | 0.39 | 0.73 | 0.08 |
| 16WA | Frontier | 1.01 | 0.40 | 0.73 | 0.09 |
| 16MD | Dynamite | 1.00 | 0.42 | 0.73 | 0.09 |
| 16CA | Starfire II | 0.99 | 0.41 | 0.73 | 0.09 |
| 16ETX | Purple Prosperity | 0.99 | 1.55 | 0.73 | 0.37 |
| 16MO | Kenland | 0.99 | 0.42 | 0.73 | 0.09 |
| 17NV | Purple Bounty | 0.98 | 1.53 | 0.73 | 0.36 |
| 16AZ | Dunn | 0.98 | 0.42 | 0.73 | 0.09 |
| 17MO | Purple Bounty | 0.98 | 1.13 | 0.73 | 0.26 |
| 16AZ | Dynamite | 0.98 | 1.84 | 0.73 | 0.43 |
| 16NV | CCS Groff | 0.97 | 0.44 | 0.72 | 0.09 |
| 16AZ | AU Robin | 0.96 | 1.83 | 0.72 | 0.43 |
| 16MD | Wildcat | 0.96 | 0.40 | 0.72 | 0.09 |
| 17NV | Whistler | 0.94 | 0.42 | 0.72 | 0.09 |
| 17AR | AU Sunup | 0.94 | 0.39 | 0.72 | 0.09 |
| 16OR | Survivor 15 | 0.93 | 1.22 | 0.72 | 0.29 |
| 16WV | Wildcat | 0.93 | 0.87 | 0.72 | 0.20 |
| 17NV | Lana | 0.92 | 0.54 | 0.71 | 0.12 |
| 17MS | Windham | 0.92 | 0.55 | 0.71 | 0.12 |
| 16KS | Frontier | 0.91 | 1.69 | 0.71 | 0.40 |
| 16AZ | Fixation | 0.90 | 1.91 | 0.71 | 0.44 |
| 17AR | Survivor 15 | 0.90 | 1.31 | 0.71 | 0.31 |
| 16ETX | Purple Bounty | 0.85 | 0.36 | 0.70 | 0.08 |
| 16KS | Survivor 15 | 0.85 | 0.41 | 0.70 | 0.09 |
| 17MS | Whistler | 0.85 | 0.41 | 0.70 | 0.09 |
| 17ETX | Frost Master | 0.84 | 0.53 | 0.70 | 0.12 |
| 16WA | AU Sunup | 0.84 | 0.37 | 0.70 | 0.08 |
| 16CA | Purple Bounty | 0.83 | 0.37 | 0.70 | 0.08 |
| 17ETX | Lana | 0.83 | 0.46 | 0.70 | 0.10 |
| 17GA | Maxum | 0.83 | 0.58 | 0.70 | 0.13 |
| 17ETX | Maxum | 0.83 | 0.50 | 0.70 | 0.12 |
| 16WV | Windham | 0.83 | 0.91 | 0.70 | 0.22 |
| 17NV | TNT | 0.82 | 0.46 | 0.69 | 0.11 |
| 16ETX | TNT | 0.82 | 1.54 | 0.69 | 0.37 |
| 17MO | Dynamite | 0.80 | 1.12 | 0.69 | 0.27 |
| 17GA | Purple Bounty | 0.80 | 0.47 | 0.69 | 0.11 |
| 16WA | Whistler | 0.79 | 1.63 | 0.69 | 0.39 |
| 16ETX | Dynamite | 0.76 | 0.38 | 0.68 | 0.09 |
| 16CA | Dynamite | 0.75 | 0.35 | 0.68 | 0.08 |
| 17NV | Kenland | 0.74 | 1.53 | 0.68 | 0.36 |
| 17NV | Fixation | 0.73 | 1.61 | 0.68 | 0.38 |
| 17MO | Fixation | 0.73 | 1.23 | 0.67 | 0.30 |
| 16AZ | Lynx | 0.73 | 0.44 | 0.67 | 0.10 |
| 17ETX | Purple Prosperity | 0.73 | 0.45 | 0.67 | 0.10 |
| 17ID | Lana | 0.70 | 0.37 | 0.67 | 0.09 |
| 17MO | Windham | 0.69 | 0.49 | 0.67 | 0.11 |
| 16NM | Survivor 15 | 0.69 | 0.39 | 0.67 | 0.09 |
| 17AR | Lynx | 0.65 | 1.29 | 0.66 | 0.31 |
| 17NV | Kentucky Pride | 0.65 | 0.36 | 0.66 | 0.09 |
| 17ETX | Purple Bounty | 0.64 | 0.47 | 0.66 | 0.11 |
| 16AZ | Cyclone II | 0.64 | 0.40 | 0.65 | 0.10 |
| 16MO | Fixation | 0.63 | 1.03 | 0.65 | 0.25 |
| 16WA | Survivor 15 | 0.63 | 1.64 | 0.65 | 0.39 |
| 16AZ | Cinammon Plus | 0.63 | 0.38 | 0.65 | 0.09 |
| 16AZ | Mammoth | 0.63 | 1.85 | 0.65 | 0.42 |
| 17NV | Freedom | 0.63 | 1.52 | 0.65 | 0.36 |
| 17MO | Freedom | 0.62 | 1.12 | 0.65 | 0.27 |
| 17NV | Mammoth | 0.62 | 0.42 | 0.65 | 0.10 |
| 16MI | Frontier | 0.62 | 3.42 | 0.65 | 0.59 |
| 16WV | Lana | 0.61 | 0.88 | 0.65 | 0.21 |
| 17MO | Contea | 0.60 | 1.14 | 0.65 | 0.28 |
| 16ID | Survivor 15 | 0.59 | 0.37 | 0.64 | 0.09 |
| 16MD | Lynx | 0.59 | 0.59 | 0.64 | 0.14 |
| 17TX | Dunn | 0.57 | 1.54 | 0.64 | 0.36 |
| 17MO | Purple Prosperity | 0.55 | 0.42 | 0.63 | 0.10 |
| 16WA | Purple Bounty | 0.55 | 0.39 | 0.63 | 0.09 |
| 17MS | Survivor 15 | 0.53 | 0.45 | 0.63 | 0.11 |
| 16NV | Purple Bounty | 0.52 | 0.48 | 0.63 | 0.12 |
| 16NY | CCS Groff | 0.52 | 0.43 | 0.63 | 0.11 |
| 17MO | Lana | 0.52 | 0.40 | 0.63 | 0.10 |
| 16MO | Contea | 0.51 | 0.92 | 0.62 | 0.23 |
| 16WA | CCS Groff | 0.50 | 0.41 | 0.62 | 0.10 |
| 17MO | Cyclone II | 0.50 | 1.12 | 0.62 | 0.27 |
| 16NV | Dixie | 0.47 | 4.31 | 0.62 | 0.60 |
| 16ETX | Freedom | 0.47 | 0.35 | 0.61 | 0.09 |
| 17NV | Cinammon Plus | 0.46 | 1.52 | 0.61 | 0.36 |
| 16AZ | Wildcat | 0.46 | 0.39 | 0.61 | 0.10 |
| 16ETX | AU Robin | 0.46 | 1.54 | 0.61 | 0.36 |
| 16MO | Freedom | 0.44 | 0.38 | 0.61 | 0.09 |
| 16WV | AU Sunup | 0.44 | 0.43 | 0.61 | 0.11 |
| 17NV | AU Sunup | 0.44 | 1.54 | 0.61 | 0.36 |
| 17MO | AU Sunup | 0.44 | 1.14 | 0.61 | 0.28 |
| 16AZ | Purple Bounty | 0.42 | 0.41 | 0.60 | 0.10 |
| 16WV | Whistler | 0.40 | 0.88 | 0.60 | 0.22 |
| 17ETX | TNT | 0.39 | 0.43 | 0.60 | 0.11 |
| 16MD | Windham | 0.38 | 0.49 | 0.59 | 0.12 |
| 17TX | Arvica 4010 | 0.37 | 1.57 | 0.59 | 0.36 |
| 17NV | Wildcat | 0.36 | 1.52 | 0.59 | 0.35 |
| 16AZ | TNT | 0.35 | 0.39 | 0.59 | 0.10 |
| 17ETX | Arvica 4010 | 0.35 | 0.38 | 0.59 | 0.10 |
| 16MO | AU Sunup | 0.34 | 0.93 | 0.58 | 0.23 |
| 16AZ | Freedom | 0.34 | 0.39 | 0.58 | 0.10 |
| 17AR | Whistler | 0.33 | 0.39 | 0.58 | 0.10 |
| 17OR | Frontier | 0.33 | 2.34 | 0.58 | 0.46 |
| 16ETX | AU Sunup | 0.30 | 0.35 | 0.57 | 0.09 |
| 16ETX | Starfire II | 0.29 | 0.37 | 0.57 | 0.09 |
| 16TX | Wildcat | 0.28 | 0.37 | 0.57 | 0.09 |
| 16ETX | Contea | 0.28 | 1.55 | 0.57 | 0.35 |
| 16MO | Lynx | 0.27 | 0.42 | 0.57 | 0.10 |
| 16OR | Frost Master | 0.27 | 1.21 | 0.57 | 0.29 |
| 17NV | Windham | 0.26 | 1.54 | 0.56 | 0.35 |
| 16WV | Survivor 15 | 0.25 | 0.90 | 0.56 | 0.22 |
| 17MO | Lynx | 0.24 | 0.45 | 0.56 | 0.11 |
| 16WA | Villana | 0.20 | 0.42 | 0.55 | 0.10 |
| 17NV | Frontier | 0.17 | 0.41 | 0.54 | 0.10 |
| 16ETX | Cyclone II | 0.17 | 1.54 | 0.54 | 0.34 |
| 17ETX | Dunn | 0.17 | 0.45 | 0.54 | 0.11 |
| 17OR | Whistler | 0.17 | 0.42 | 0.54 | 0.10 |
| 16MO | Windham | 0.16 | 0.93 | 0.54 | 0.22 |
| 16WA | Lana | 0.14 | 0.38 | 0.54 | 0.09 |
| 16ID | Contea | 0.14 | 0.35 | 0.54 | 0.09 |
| 16MO | Survivor 15 | 0.12 | 0.42 | 0.53 | 0.10 |
| 16AZ | Contea | 0.11 | 0.36 | 0.53 | 0.09 |
| 16WA | TNT | 0.11 | 0.37 | 0.53 | 0.09 |
| 16NY | Fixation | 0.10 | 0.39 | 0.53 | 0.10 |
| 17OR | Frost Master | 0.10 | 0.38 | 0.53 | 0.09 |
| 16AZ | CCS Groff | 0.07 | 0.39 | 0.52 | 0.10 |
| 16KS | Dunn | 0.07 | 0.42 | 0.52 | 0.10 |
| 16MO | Starfire II | 0.06 | 0.41 | 0.52 | 0.10 |
| 16MO | Lana | 0.05 | 0.46 | 0.51 | 0.11 |
| 16MO | Mammoth | 0.02 | 0.42 | 0.51 | 0.10 |
| 16TX | Starfire II | 0.02 | 0.44 | 0.51 | 0.11 |
| 16MO | Wildcat | 0.01 | 0.39 | 0.50 | 0.10 |
| 16KS | Windham | 0.01 | 0.39 | 0.50 | 0.10 |
| 16NV | Survivor 15 | 0.00 | 0.43 | 0.50 | 0.11 |
| 16WV | Lynx | -0.01 | 0.88 | 0.50 | 0.21 |
| 16TX | Cyclone II | -0.01 | 0.39 | 0.50 | 0.10 |
| 16NV | Lynx | -0.01 | 0.52 | 0.50 | 0.13 |
| 16AZ | AU Sunup | -0.04 | 0.36 | 0.49 | 0.09 |
| 16MO | Cyclone II | -0.05 | 0.39 | 0.49 | 0.10 |
| 16NY | TNT | -0.06 | 0.49 | 0.48 | 0.12 |
| 17MO | AU Sunrise | -0.06 | 0.48 | 0.48 | 0.12 |
| 16ID | Frontier | -0.11 | 0.36 | 0.47 | 0.09 |
| 16OR | Whistler | -0.11 | 0.42 | 0.47 | 0.10 |
| 16OR | Frontier | -0.12 | 1.29 | 0.47 | 0.27 |
| 16ETX | Wildcat | -0.12 | 0.36 | 0.47 | 0.09 |
| 16NV | Dynamite | -0.14 | 4.31 | 0.46 | 0.45 |
| 17AR | Frontier | -0.15 | 1.37 | 0.46 | 0.28 |
| 16AZ | Survivor 15 | -0.15 | 1.85 | 0.46 | 0.34 |
| 16NV | AU Robin | -0.16 | 4.31 | 0.46 | 0.45 |
| 16MO | Whistler | -0.17 | 0.41 | 0.46 | 0.10 |
| 16NY | Dixie | -0.17 | 0.41 | 0.46 | 0.10 |
| 16TX | Mammoth | -0.17 | 0.44 | 0.46 | 0.11 |
| 16MI | Lana | -0.19 | 0.44 | 0.45 | 0.11 |
| 16ETX | Cinammon Plus | -0.21 | 0.37 | 0.45 | 0.09 |
| 17AR | Windham | -0.24 | 0.42 | 0.44 | 0.10 |
| 16ETX | Villana | -0.26 | 0.37 | 0.44 | 0.09 |
| 17WA | Windham | -0.27 | 0.41 | 0.43 | 0.10 |
| 17CO | Kenland | -0.27 | 0.48 | 0.43 | 0.11 |
| 16MS | Maxum | -0.30 | 0.57 | 0.43 | 0.13 |
| 16NV | AU Sunrise | -0.31 | 0.36 | 0.42 | 0.09 |
| 17NV | Survivor 15 | -0.32 | 1.54 | 0.42 | 0.29 |
| 16NV | Freedom | -0.32 | 4.31 | 0.42 | 0.41 |
| 16MO | Cinammon Plus | -0.33 | 0.40 | 0.42 | 0.09 |
| 16WA | Purple Prosperity | -0.33 | 0.41 | 0.42 | 0.09 |
| 16NV | Contea | -0.34 | 4.31 | 0.41 | 0.41 |
| 17WA | Survivor 15 | -0.35 | 0.39 | 0.41 | 0.09 |
| 16NY | AU Robin | -0.36 | 0.39 | 0.41 | 0.09 |
| 16AR | Frost Master | -0.36 | 0.40 | 0.41 | 0.09 |
| 16NM | Dunn | -0.40 | 3.57 | 0.40 | 0.38 |
| 16WV | Frost Master | -0.42 | 0.88 | 0.40 | 0.18 |
| 16MO | Frost Master | -0.43 | 0.46 | 0.39 | 0.10 |
| 16NY | Kentucky Pride | -0.44 | 1.22 | 0.39 | 0.23 |
| 16AZ | AU Sunrise | -0.44 | 0.35 | 0.39 | 0.08 |
| 16AZ | Kentucky Pride | -0.45 | 0.36 | 0.39 | 0.08 |
| 16NV | Cyclone II | -0.45 | 4.31 | 0.39 | 0.38 |
| 16MO | AU Robin | -0.46 | 0.36 | 0.39 | 0.08 |
| 16ID | AU Sunup | -0.49 | 0.36 | 0.38 | 0.08 |
| 16NV | AU Sunup | -0.51 | 4.32 | 0.38 | 0.37 |
| 16WA | Maxum | -0.51 | 0.47 | 0.38 | 0.10 |
| 16ETX | Kenland | -0.52 | 0.42 | 0.37 | 0.09 |
| 16NY | Purple Prosperity | -0.53 | 1.21 | 0.37 | 0.22 |
| 17CO | Windham | -0.55 | 0.96 | 0.37 | 0.18 |
| 16AZ | Dixie | -0.57 | 0.36 | 0.36 | 0.08 |
| 16NY | AU Sunrise | -0.60 | 1.23 | 0.35 | 0.22 |
| 16ETX | Survivor 15 | -0.65 | 1.56 | 0.34 | 0.24 |
| 16NV | Windham | -0.69 | 4.32 | 0.33 | 0.33 |
| 16KS | Lynx | -0.70 | 0.41 | 0.33 | 0.08 |
| 17MO | Survivor 15 | -0.71 | 0.46 | 0.33 | 0.09 |
| 17CO | Purple Bounty | -0.72 | 0.39 | 0.33 | 0.08 |
| 17CO | CCS Groff | -0.72 | 0.36 | 0.33 | 0.07 |
| 17MS | Maxum | -0.73 | 2.13 | 0.32 | 0.27 |
| 16ID | Frost Master | -0.74 | 0.43 | 0.32 | 0.09 |
| 16ID | Lynx | -0.75 | 0.46 | 0.32 | 0.09 |
| 16WA | Arvica 4010 | -0.79 | 0.39 | 0.31 | 0.08 |
| 16NY | Purple Bounty | -0.79 | 0.45 | 0.31 | 0.09 |
| 16ETX | Mammoth | -0.81 | 0.37 | 0.31 | 0.07 |
| 16WV | Frontier | -0.81 | 0.99 | 0.31 | 0.17 |
| 17CO | Cyclone II | -0.81 | 0.38 | 0.31 | 0.07 |
| 16KS | Maxum | -0.81 | 0.45 | 0.31 | 0.09 |
| 16AZ | Frost Master | -0.81 | 1.84 | 0.31 | 0.24 |
| 17MO | AU Robin | -0.84 | 0.47 | 0.30 | 0.09 |
| 16NY | Contea | -0.87 | 0.38 | 0.30 | 0.07 |
| 17CO | TNT | -0.88 | 0.36 | 0.29 | 0.07 |
| 17TX | Maxum | -0.89 | 0.47 | 0.29 | 0.09 |
| 16ETX | Lynx | -0.90 | 1.55 | 0.29 | 0.21 |
| 17MO | Whistler | -0.91 | 0.50 | 0.29 | 0.09 |
| 17AR | Frost Master | -0.92 | 0.47 | 0.28 | 0.09 |
| 16NM | Frost Master | -0.93 | 0.53 | 0.28 | 0.09 |
| 16NV | Dunn | -0.96 | 0.47 | 0.28 | 0.08 |
| 17CO | Villana | -0.98 | 0.38 | 0.27 | 0.07 |
| 17NV | Contea | -1.03 | 0.47 | 0.26 | 0.08 |
| 17CO | Cinammon Plus | -1.08 | 0.36 | 0.25 | 0.06 |
| 17CO | Kentucky Pride | -1.15 | 1.59 | 0.24 | 0.18 |
| 17MS | Dunn | -1.15 | 2.16 | 0.24 | 0.20 |
| 16AZ | Frontier | -1.20 | 1.90 | 0.23 | 0.19 |
| 17CO | Purple Prosperity | -1.25 | 1.58 | 0.22 | 0.17 |
| 16NY | Freedom | -1.27 | 0.46 | 0.22 | 0.07 |
| 16ETX | Frost Master | -1.31 | 1.55 | 0.21 | 0.16 |
| 16NY | Cyclone II | -1.35 | 1.21 | 0.21 | 0.13 |
| 17MS | Arvica 4010 | -1.35 | 2.18 | 0.21 | 0.18 |
| 16OR | Lynx | -1.37 | 0.41 | 0.20 | 0.06 |
| 17MO | Frontier | -1.38 | 1.21 | 0.20 | 0.13 |
| 16NM | Lana | -1.39 | 1.69 | 0.20 | 0.16 |
| 16NY | Mammoth | -1.40 | 1.23 | 0.20 | 0.13 |
| 16NY | AU Sunup | -1.41 | 1.23 | 0.20 | 0.13 |
| 16MD | Frost Master | -1.47 | 0.56 | 0.19 | 0.07 |
| 16MO | Frontier | -1.47 | 1.01 | 0.19 | 0.11 |
| 17NV | Frost Master | -1.50 | 0.47 | 0.18 | 0.06 |
| 17NV | AU Sunrise | -1.52 | 0.46 | 0.18 | 0.06 |
| 16NY | Windham | -1.59 | 1.23 | 0.17 | 0.11 |
| 17CO | Starfire II | -1.60 | 0.51 | 0.17 | 0.06 |
| 17ID | Dunn | -1.65 | 1.66 | 0.16 | 0.13 |
| 16NY | Cinammon Plus | -1.73 | 0.46 | 0.15 | 0.05 |
| 17MO | Frost Master | -1.76 | 0.56 | 0.15 | 0.06 |
| 17CO | AU Robin | -1.78 | 1.57 | 0.14 | 0.11 |
| 16NY | Lana | -1.81 | 1.21 | 0.14 | 0.09 |
| 17CO | Fixation | -1.83 | 1.66 | 0.14 | 0.11 |
| 16MI | Maxum | -1.86 | 3.40 | 0.13 | 0.13 |
| 16MS | Dunn | -1.87 | 1.31 | 0.13 | 0.09 |
| 16NM | Lynx | -1.87 | 0.80 | 0.13 | 0.07 |
| 16NV | Frost Master | -1.94 | 4.31 | 0.13 | 0.12 |
| 17CO | Contea | -1.96 | 1.59 | 0.12 | 0.10 |
| 17NV | Maxum | -1.97 | 0.46 | 0.12 | 0.04 |
| 16NV | Kentucky Pride | -1.99 | 0.38 | 0.12 | 0.03 |
| 17GA | Dunn | -1.99 | 1.62 | 0.12 | 0.09 |
| 16NY | Wildcat | -2.00 | 0.42 | 0.12 | 0.04 |
| 16NY | Whistler | -2.02 | 1.21 | 0.12 | 0.08 |
| 17WA | Frost Master | -2.06 | 0.65 | 0.11 | 0.05 |
| 16NY | Starfire II | -2.06 | 0.48 | 0.11 | 0.04 |
| 16MS | Arvica 4010 | -2.07 | 1.35 | 0.11 | 0.08 |
| 16NY | Frontier | -2.08 | 0.49 | 0.11 | 0.04 |
| 17WA | Dixie | -2.10 | 0.46 | 0.11 | 0.04 |
| 17CO | Mammoth | -2.11 | 1.59 | 0.11 | 0.08 |
| 16ID | Whistler | -2.12 | 0.53 | 0.11 | 0.04 |
| 17CO | AU Sunup | -2.12 | 1.59 | 0.11 | 0.08 |
| 16KS | Arvica 4010 | -2.19 | 1.70 | 0.10 | 0.08 |
| 17GA | Arvica 4010 | -2.19 | 1.65 | 0.10 | 0.08 |
| 17CO | Dynamite | -2.21 | 0.36 | 0.10 | 0.03 |
| 17CO | Wildcat | -2.21 | 1.58 | 0.10 | 0.08 |
| 16MI | Dunn | -2.28 | 3.42 | 0.09 | 0.09 |
| 16TX | Frontier | -2.32 | 1.04 | 0.09 | 0.06 |
| 16TX | Maxum | -2.33 | 1.87 | 0.09 | 0.07 |
| 17NV | Lynx | -2.36 | 0.76 | 0.09 | 0.04 |
| 17WA | Lana | -2.40 | 0.64 | 0.08 | 0.04 |
| 16ID | Windham | -2.42 | 0.59 | 0.08 | 0.03 |
| 17CO | Dixie | -2.46 | 0.58 | 0.08 | 0.03 |
| 16NY | Dynamite | -2.48 | 0.42 | 0.08 | 0.03 |
| 17AR | Maxum | -2.63 | 1.31 | 0.07 | 0.05 |
| 16NY | Kenland | -2.65 | 0.52 | 0.07 | 0.03 |
| 17WA | Whistler | -2.67 | 0.71 | 0.06 | 0.03 |
| 17CO | Whistler | -2.73 | 1.58 | 0.06 | 0.05 |
| 16TX | Dunn | -2.76 | 1.90 | 0.06 | 0.05 |
| 17OR | Arvica 4010 | -2.77 | 2.35 | 0.06 | 0.05 |
| 16ETX | Fixation | -2.82 | 0.80 | 0.06 | 0.03 |
| 17CO | Freedom | -2.86 | 0.55 | 0.05 | 0.02 |
| 16NY | Lynx | -2.87 | 1.01 | 0.05 | 0.03 |
| 17CO | Survivor 15 | -2.89 | 1.59 | 0.05 | 0.04 |
| 16ETX | Frontier | -2.89 | 1.08 | 0.05 | 0.03 |
| 16TX | Arvica 4010 | -2.96 | 1.93 | 0.05 | 0.04 |
| 16ID | Maxum | -2.98 | 2.85 | 0.05 | 0.05 |
| 16MD | Frontier | -3.01 | 0.86 | 0.05 | 0.03 |
| 16OR | Dunn | -3.02 | 1.27 | 0.05 | 0.03 |
| 17CO | AU Sunrise | -3.05 | 0.77 | 0.05 | 0.02 |
| 17OR | Maxum | -3.05 | 0.65 | 0.05 | 0.02 |
| 17CO | Lynx | -3.14 | 1.58 | 0.04 | 0.03 |
| 16NY | Survivor 15 | -3.14 | 0.88 | 0.04 | 0.02 |
| 16OR | Arvica 4010 | -3.22 | 1.31 | 0.04 | 0.03 |
| 17AR | Arvica 4010 | -3.25 | 1.39 | 0.04 | 0.03 |
| 16NY | Frost Master | -3.28 | 1.01 | 0.04 | 0.02 |
| 16WV | Maxum | -3.29 | 0.90 | 0.04 | 0.02 |
| 16WA | Dunn | -3.32 | 1.68 | 0.03 | 0.03 |
| 17WA | Freedom | -3.42 | 0.57 | 0.03 | 0.01 |
| 17WA | Purple Prosperity | -3.45 | 3.17 | 0.03 | 0.03 |
| 17WA | AU Sunrise | -3.52 | 3.17 | 0.03 | 0.03 |
| 17CO | Frost Master | -3.55 | 1.58 | 0.03 | 0.02 |
| 17WA | Kentucky Pride | -3.60 | 0.45 | 0.03 | 0.01 |
| 16ID | Arvica 4010 | -3.60 | 2.89 | 0.03 | 0.03 |
| 17WA | TNT | -3.62 | 3.16 | 0.03 | 0.02 |
| 16AZ | Maxum | -3.68 | 1.85 | 0.02 | 0.02 |
| 16WV | Dunn | -3.71 | 0.97 | 0.02 | 0.01 |
| 17WA | Purple Bounty | -3.78 | 3.17 | 0.02 | 0.02 |
| 16NV | Mammoth | -3.85 | 0.98 | 0.02 | 0.01 |
| 16OR | Maxum | -3.87 | 0.82 | 0.02 | 0.01 |
| 16WV | Arvica 4010 | -3.91 | 1.01 | 0.02 | 0.01 |
| 17CO | Frontier | -3.94 | 1.65 | 0.02 | 0.02 |
| 17WA | AU Robin | -3.98 | 3.16 | 0.02 | 0.02 |
| 17WV | Kentucky Pride | -4.02 | 4.56 | 0.02 | 0.02 |
| 17WA | Fixation | -4.03 | 3.20 | 0.02 | 0.02 |
| 17WV | Villana | -4.11 | 4.56 | 0.02 | 0.02 |
| 17AR | Dunn | -4.15 | 0.92 | 0.02 | 0.01 |
| 17OR | Dunn | -4.16 | 0.72 | 0.02 | 0.01 |
| 16ETX | Maxum | -4.18 | 1.56 | 0.02 | 0.01 |
| 16MI | Frost Master | -4.18 | 1.35 | 0.02 | 0.01 |
| 17WA | Cyclone II | -4.27 | 3.16 | 0.01 | 0.01 |
| 16AZ | Arvica 4010 | -4.30 | 1.91 | 0.01 | 0.01 |
| 17ID | Maxum | -4.31 | 0.83 | 0.01 | 0.01 |
| 16MD | Maxum | -4.32 | 1.01 | 0.01 | 0.01 |
| 17WA | AU Sunup | -4.33 | 3.17 | 0.01 | 0.01 |
| 17MO | Maxum | -4.39 | 0.92 | 0.01 | 0.01 |
| 17WA | Wildcat | -4.41 | 3.16 | 0.01 | 0.01 |
| 17ID | Arvica 4010 | -4.43 | 0.88 | 0.01 | 0.01 |
| 17MO | Arvica 4010 | -4.48 | 1.23 | 0.01 | 0.01 |
| 16MO | Maxum | -4.51 | 0.74 | 0.01 | 0.01 |
| 16ETX | Dunn | -4.60 | 1.60 | 0.01 | 0.01 |
| 17OR | Lynx | -4.62 | 0.91 | 0.01 | 0.01 |
| 17WV | Dynamite | -4.63 | 4.56 | 0.01 | 0.01 |
| 16MD | Dunn | -4.63 | 1.04 | 0.01 | 0.01 |
| 17WV | Starfire II | -4.69 | 4.56 | 0.01 | 0.01 |
| 17WV | Kenland | -4.69 | 4.56 | 0.01 | 0.01 |
| 16MO | Dunn | -4.72 | 0.83 | 0.01 | 0.00 |
| 16MD | Arvica 4010 | -4.74 | 1.07 | 0.01 | 0.01 |
| 16NV | Kenland | -4.80 | 1.82 | 0.01 | 0.01 |
| 16ETX | Arvica 4010 | -4.80 | 1.63 | 0.01 | 0.01 |
| 16ID | Lana | -4.81 | 1.11 | 0.01 | 0.01 |
| 16MO | Arvica 4010 | -4.87 | 0.88 | 0.01 | 0.00 |
| 17MO | Dunn | -4.91 | 0.91 | 0.01 | 0.00 |
| 17WV | Cyclone II | -4.93 | 4.56 | 0.01 | 0.01 |
| 16NV | Frontier | -4.95 | 2.25 | 0.01 | 0.01 |
| 16NV | Starfire II | -4.96 | 1.79 | 0.01 | 0.01 |
| 17WV | AU Sunup | -4.99 | 4.56 | 0.01 | 0.01 |
| 17NV | Dunn | -5.15 | 1.13 | 0.01 | 0.00 |
| 16AR | Whistler | -5.17 | 1.34 | 0.01 | 0.00 |
| 17NV | Arvica 4010 | -5.31 | 1.16 | 0.00 | 0.00 |
| 17WA | Lynx | -5.34 | 3.16 | 0.00 | 0.00 |
| 17WV | Lana | -5.39 | 4.56 | 0.00 | 0.00 |
| 17CO | Lana | -5.45 | 0.82 | 0.00 | 0.00 |
| 16ID | Dunn | -5.51 | 1.61 | 0.00 | 0.00 |
| 16AR | Maxum | -5.52 | 1.69 | 0.00 | 0.00 |
| 17WV | Whistler | -5.60 | 4.56 | 0.00 | 0.00 |
| 17WA | Villana | -5.66 | 1.74 | 0.00 | 0.00 |
| 16AR | Dunn | -5.69 | 1.73 | 0.00 | 0.00 |
| 16NV | Fixation | -5.70 | 1.69 | 0.00 | 0.00 |
| 16NY | Maxum | -5.75 | 1.20 | 0.00 | 0.00 |
| 17WV | Survivor 15 | -5.75 | 4.56 | 0.00 | 0.00 |
| 16MI | Arvica 4010 | -5.86 | 1.59 | 0.00 | 0.00 |
| 16NV | Cinammon Plus | -5.98 | 1.68 | 0.00 | 0.00 |
| 17WA | Contea | -5.99 | 1.86 | 0.00 | 0.00 |
| 16NV | Wildcat | -6.00 | 1.69 | 0.00 | 0.00 |
| 17WV | Lynx | -6.01 | 4.56 | 0.00 | 0.00 |
| 17WA | CCS Groff | -6.09 | 1.58 | 0.00 | 0.00 |
| 17WA | Kenland | -6.12 | 1.77 | 0.00 | 0.00 |
| 17WA | Starfire II | -6.14 | 1.77 | 0.00 | 0.00 |
| 17WA | Frontier | -6.14 | 3.20 | 0.00 | 0.00 |
| 16NY | Dunn | -6.15 | 1.26 | 0.00 | 0.00 |
| 16NY | Arvica 4010 | -6.35 | 1.29 | 0.00 | 0.00 |
| 17WA | Mammoth | -6.36 | 1.79 | 0.00 | 0.00 |
| 17WV | AU Sunrise | -6.40 | 2.48 | 0.00 | 0.00 |
| 17WV | Frost Master | -6.42 | 4.56 | 0.00 | 0.00 |
| 17CO | Maxum | -6.42 | 1.59 | 0.00 | 0.00 |
| 17WV | Fixation | -6.42 | 2.71 | 0.00 | 0.00 |
| 16AR | Arvica 4010 | -6.46 | 1.64 | 0.00 | 0.00 |
| 17WV | AU Robin | -6.73 | 2.53 | 0.00 | 0.00 |
| 17WV | Frontier | -6.81 | 4.58 | 0.00 | 0.00 |
| 17CO | Dunn | -6.84 | 1.63 | 0.00 | 0.00 |
| 17WA | Dynamite | -6.90 | 1.57 | 0.00 | 0.00 |
| 17WV | Contea | -6.90 | 2.54 | 0.00 | 0.00 |
| 17WV | Wildcat | -6.97 | 2.62 | 0.00 | 0.00 |
| 17WA | Cinammon Plus | -7.10 | 1.60 | 0.00 | 0.00 |
| 17WV | Dixie | -7.15 | 2.18 | 0.00 | 0.00 |
| 17WV | Freedom | -7.34 | 2.37 | 0.00 | 0.00 |
| 17CO | Arvica 4010 | -7.42 | 1.38 | 0.00 | 0.00 |
| 17WV | Cinammon Plus | -7.51 | 2.36 | 0.00 | 0.00 |
| 17WV | Windham | -7.55 | 2.42 | 0.00 | 0.00 |
| 17WV | Mammoth | -7.57 | 2.35 | 0.00 | 0.00 |
| 17WA | Maxum | -8.66 | 3.11 | 0.00 | 0.00 |
| 17WA | Dunn | -9.04 | 3.19 | 0.00 | 0.00 |
| 17WA | Arvica 4010 | -9.24 | 3.21 | 0.00 | 0.00 |
| 17WV | Maxum | -9.29 | 4.56 | 0.00 | 0.00 |
| 17WV | Dunn | -9.82 | 4.30 | 0.00 | 0.00 |
| 17WV | Arvica 4010 | -9.91 | 4.59 | 0.00 | 0.00 |
